# Comparative analysis of MACROD1, MACROD2 and TARG1 expression, localisation and interactome

**DOI:** 10.1038/s41598-020-64623-y

**Published:** 2020-05-19

**Authors:** R. Žaja, G. Aydin, B. E. Lippok, R. Feederle, B. Lüscher, K.L.H. Feijs

**Affiliations:** 1 Institute of Biochemistry and Molecular Biology, RWTH Aachen University, Pauwelsstrasse 30, 52074 Aachen, Germany; 2 Monoclonal Antibody Core Facility, Institute for Diabetes and Obesity, Helmholtz Zentrum München, German Research Center for Environmental Health, Neuherberg, Germany

**Keywords:** Post-translational modifications, Hydrolases, Post-translational modifications

## Abstract

The posttranslational modification ADP-ribosylation is involved in many cellular processes, with distinct roles for poly- and mono(ADP-ribosyl)ation (PAR- and MARylation, respectively). Reversibility of intracellular MARylation was demonstrated with the discovery of MACROD1, MACROD2 and TARG1, three macrodomain-containing enzymes capable of reversing MARylation of proteins and RNA. While the three enzymes have identical activities *in vitro*, their roles in cells are unclear and published data are partially contradictory, possibly due to a lack of validated reagents. We developed monoclonal antibodies to study these proteins and analysed their tissue distribution and intracellular localisation. MACROD1 is most prevalent in mitochondria of skeletal muscle, MACROD2 localises to nucleo- and cytoplasm and is found so far only in neuroblastoma cells, whereas the more ubiquitously expressed TARG1 is present in nucleoplasm, nucleolus and stress granules. Loss of MACROD1 or loss of TARG1 leads to disruption of mitochondrial or nucleolar morphology, respectively, hinting at their importance for these organelles. To start elucidating the underlying mechanisms, we have mapped their interactomes using BioID. The cellular localisation of interactors supports the mitochondrial, nucleolar and stress granule localisation of MACROD1 and TARG1, respectively. Gene ontology analysis suggests an involvement of MACROD1 and TARG1 in RNA metabolism in their respective compartments. The detailed description of the hydrolases’ expression, localisation and interactome presented here provides a solid basis for future work addressing their physiological function in more detail.

## Introduction

The modification of proteins with ADP-ribose, known as ADP-ribosylation, was discovered over 50 years ago^1^. The formation of polymers of ADP-ribose, poly-ADP-ribosylation or PARylation, is known to be involved in many cellular processes. Most notably in the DNA damage response, but also in for example the regulation of chromatin structure, transcription and RNA processing^2^. PARylation is performed by certain enzymes of the PARP protein family, which is also known as ARTD protein family^3^. The majority of enzymes in this family is capable of transferring only one ADP-ribose moiety (MARylation)^4,5^. One of the most recently emerging functions for MARylation is in the host-viral interaction, as expression of several of the MARylating PARP/ARTD family members is induced upon interferon stimulation and was reported to limit viral replication^6,7^. MARylation by these PARPs may be counteracted by viral macrodomain proteins, some of which are hydrolases reversing MARylation^8–10^. PARP12/ARTD12 in return appears to be capable of modifying non-structural viral proteins, indirectly leading to their ubiquitination and subsequent degradation, thereby limiting viral replication^11^. The anti-viral response is not the only function of MARylation, as MARylation by PARP10, for example, was reported to be involved in a wide range of processes, such as NF-κB signalling^12^, aurora kinase A and GSK3β activity^13,14^, mitochondrial function^15^ and replication stress^16^. The recent development of MARylation detection reagents may assist further investigation of the physiological function of the modification^17–19^, which was not possible before due to lack of specific tools.

Like other PTMs, MARylation is fully reversible. In 2005, a protein domain referred to as macrodomain was described as ADP-ribose binding module^20^; first recognised as binder of PARylated proteins^21^, later also as binding module for MARylated proteins^22^. Three macrodomain-containing proteins, MACROD1, MACROD2 and TARG1 were reported to have hydrolase activity: first in deacetylating O-acetyl-ADP-ribose (OAADPR)^23^, next in removing ADP-ribose from modified proteins^24–27^ and lastly in removing ADP-ribose from MARylated DNA or RNA^28,29^. These mostly biochemical studies have not addressed their function in cells, leaving it unclear which of these activities is physiologically relevant. At a first glance contradicting reports describe MACROD1 (also known as leukaemia-related protein 16 or LRP16) as nuclear^30,31^ or as mitochondrial protein^31–33^, although to date only nuclear functions have been described, for example as counteracting PARP7/ARTD14^31,34^, as activator of NF-κB signalling^35^, androgen receptor signalling^36^, and oestrogen signalling^37^. A potential role for MACROD1 in mitochondria has not been confirmed nor studied as yet. MACROD2 was identified as a protein which can shuttle from the nucleus to the cytoplasm after DNA damage, dependent on phosphorylation by ATM^38^. A mutation in TARG1 was found in patients with a severe neurodegenerative phenotype, although the underlying mechanism is unclear^26^. Overexpressed TARG1 resides in both nucleoli and nucleoplasm, compartments between which it can shuttle^39^. Furthermore, several reports link MACROD1 or MACROD2 to tumorigenesis, although none have addressed potential underlying mechanisms^40–42^. Gene alterations occur in 0.9%, 2.6% and 1%, for MACROD1, MACROD2 and TARG1, respectively, of over a thousand patient samples in cBioPortal’s curated dataset, making mutations in these genes quite rare^43^.

Scattered bits of information thus hint that MACROD1, MACROD2 and TARG1 have unique functions in normal cell physiology and potentially in pathologies such as neurodegeneration and cancer, however, these functions remain barely studied. In this work we aim to provide a more comprehensive overview of the hydrolases’ expression, localisation and interactome and thereby provide a solid basis for future research.

## Results

### MACROD1, MACROD2 and TARG1 are differentially expressed

**Figure 1:**
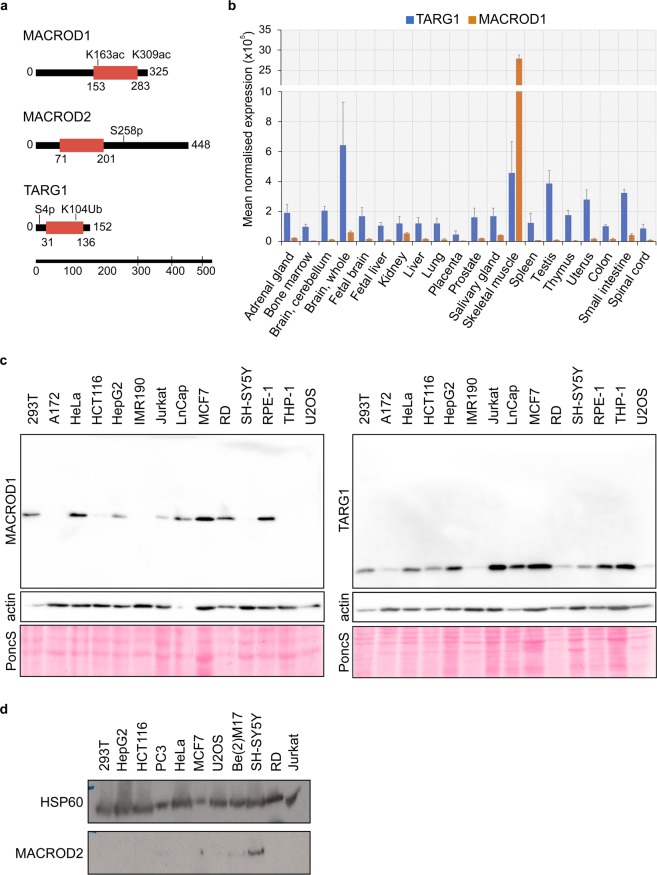
MACROD1, MACROD2 and TARG1 are differentially expressed. **(a)** Schematic representation of MACROD1, MACROD2 and TARG1 protein architecture based on SMART/PFAM domain predictions which were generated with a profile hidden Markov model using HMMER^71^. Modification sites were added when found in at least three high-throughput screens according to Phosphosite.org accessed in 2019. **(b)** *MACROD1* and *TARG1* expression were measured in an RNA tissue library (ClonTech) and displayed as fold expression over *GUS* expression. **(c)** Lysates were generated from indicated cell lines and analysed on Western Blots. MACROD1 (monoclonal antibody 28C11) and TARG1 (monoclonal antibody 3A5) were detected first on the whole blots, followed by detection of actin as loading control. A section of the Ponceau staining is also shown. **(d)** Lysates were generated from indicated cell lines and analysed by Western Blotting using a monoclonal MACROD2 antibody (18D12). To detect MACROD2, the blot was incubated with a high-sensitivity substrate and exposed to film overnight. HSP60 was detected afterwards as loading control. The whole blots are displayed in the Supplementary Figures. PoncS = Ponceau S staining.

The biochemical activities of MACROD1, MACROD2 and TARG1 are highly similar, but to date it is not known which of their substrates are essential for their cellular function. Their secondary and tertiary structures do not unveil any clues about their potential cell physiological roles, as apart from the macrodomains no other recognisable domains are present in these relatively small proteins (Fig. 1a). High-throughput screens have identified a number of posttranslational modifications (indicated in Fig. 1a)^44^, but none of these have been further analysed. Specific functions are likely to be regulated either by restricted intracellular localisation, tissue-specific expression and protein interactions. A limited number of papers have studied these proteins, with partially contradictory findings. MACROD1 for example has been reported to reside in the nucleus, the cytoplasm and/or mitochondria (Table 1).

**Table 1:** MACROD1, MACROD2 and TARG1 nomenclature and predicted/reported localisation.

Protein name	Gene name	Localisation UNIPROT	Localisation Human Protein Atlas	Localisation observed
MACROD1;LRP16	*MACROD1*	Nucleus	Nucleoplasm^45^	Nucleus; cytoplasm (GFP-MACROD1)^30^ Mitochondria (MACROD1-GFP)^32,33^ Mitochondria; nucleus (flag-MACROD1)^31^
MACROD2; C20orf133	*MACROD2*	Nucleus	Nucleoplasm; nucleoli^45^	Nucleus; cytoplasm (GFP-MACROD2)^32,38^
TARG1;C6orf130	*OARD1*	Nucleus	Nucleoplasm; nucleoli^45^	Nucleoplasm; nucleoli (GFP-TARG1)^26,39^

Table 1 summarises the commonly used protein and gene names of the three hydrolases, as well as their predicted and thus far observed intracellular localisations.

To determine which of these previous findings we can verify, we decided to test their expression and localisation. We first analysed their mRNA levels across a panel of 20 different tissues and found distinct expression levels (Fig. 1b). These are mostly consistent with RNA-seq data from the Genotype-Tissue Expression (GTEx) project^46^ (Supplementary Fig. S1a). *MACROD1* shows the highest overall mRNA expression, with striking enrichment in skeletal muscle, which fits with the expression of *MACROD1* in heart tissue reported previously^47^. *MACROD1* expression closely follows *HSP1* expression, which encodes for the mitochondrial matrix protein HSP60 (Supplementary Fig. S1b). *OARD1* (encoding TARG1) has a broad tissue distribution in the RNA-seq datasets. *MACROD2* is expressed at a low level in most tissues, with the exception of Epstein-Barr transformed lymphocytes and could not be reliably detected using our primers in the tissue RNA set (data not shown). Because mRNA and protein levels do not always correlate, we next analysed protein expression. We tested a MACROD1 antibody used before^33^, but found that many unspecific bands are present in Western Blot (antibody Abcam122688 in Supplementary Fig. S2a). Another MACROD1 antibody used in earlier publications is not commercially available^37^. This is also true for the MACROD2 polyclonal antibody #494-7^38^, which shows staining disagreeing with the results obtained with the commercial polyclonal antibody HPA049076^45,48^. This latter antibody has been discontinued by the company. Despite the poor characterisation of this now retracted MACROD2 antibody, it has for example been used to correlate protein expression to response to chemotherapy in patients with colon cancer^49^. We listed all antibodies available and have found that either no whole blots are shown to demonstrate specificity or that unspecific bands are present (Supplementary Table S1).

Therefore, we decided to generate monoclonal antibodies against recombinant proteins, performed extensive negative and positive selection and chose the antibodies, which gave the strongest signal in Western Blot on the recombinant proteins. For MacroD1 we selected two antibodies, termed 28C11 and 25E9; the 28C11 antibody results in the cleanest blots without additional bands. The 25E9 antibody gives a slightly stronger signal in Western Blot, but also results in some unspecific bands (Supplementary Fig. S2a). We next generated a panel of lysates from commonly used cell lines and tested the hydrolases’ expression using the 28C11 antibody for MacroD1, 18D12 for MacroD2 and 3A5 for TARG1. The data correlate well with the human RNA data, with TARG1 being most ubiquitously expressed, MACROD1 more specifically enriched in a number of cell lines, amongst which the rhabdomyosarcoma cell line RD and also breast cancer line MCF7 (Fig. 1c). For MACROD2, initially we did not see any signal and therefore loaded double amounts of lysate. Under these conditions, protein bands become visible in a few cell lines, most notably SH-SY5Y, at around 60 kDa (Fig. 1d). This corresponds to an earlier siRNA experiment with MACROD2, where a protein species of around 60 kDa disappeared upon knockdown^38^. This is larger than the predicted molecular weight of the canonical MACROD2 isoform and might represent effects of particular amino acid sequences or posttranslational modification(s). A recent study revealed specific expression of *MacroD2* in cortical and hippocampal neurons in the mouse brain^50^, agreeing with our findings of relatively high MACROD2 expression in human neuroblastoma cells. Together, these data show that the three hydrolases are expressed in different tissues. MACROD1 may have a specific function in skeletal muscle and MACROD2 in the brain, whereas the expression and thereby most likely also function of TARG1 appears more ubiquitous.

### MACROD1, MACROD2 and TARG localise to different intracellular compartments

**Figure 2:**
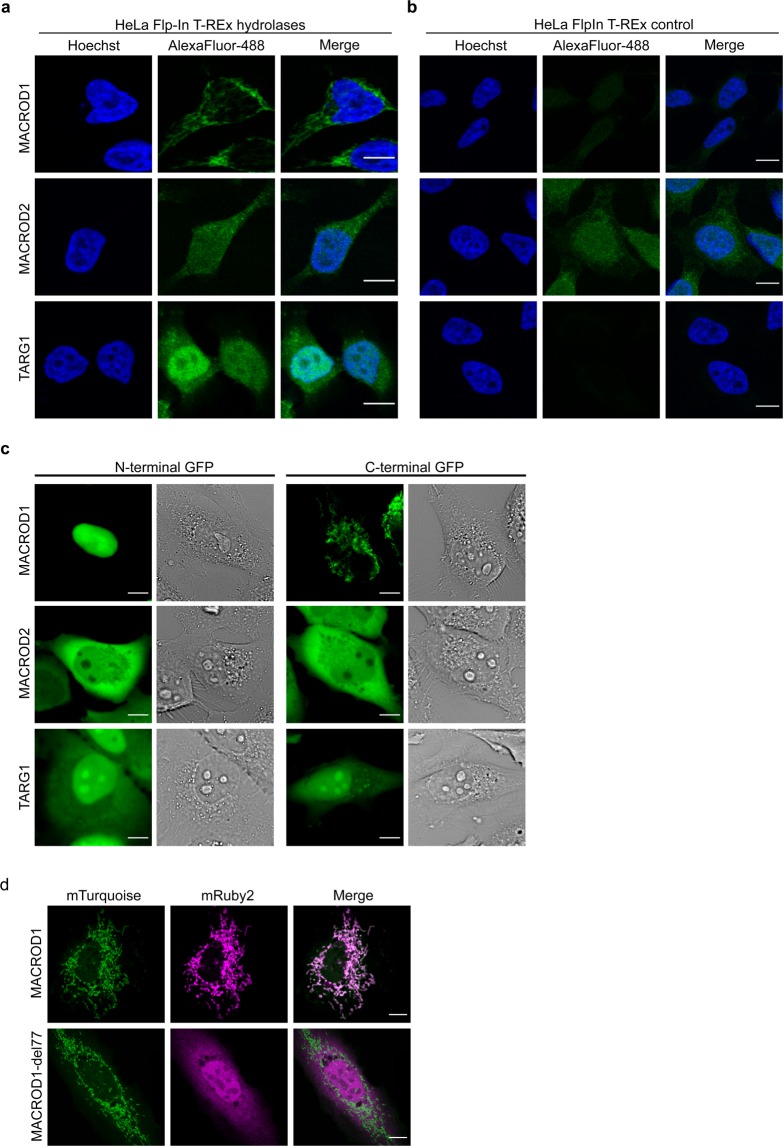
MACROD1, MACROD2 and TARG1 are differentially localised. **(a)** HeLa Flp-In T-REx cells overexpressing MACROD1, MACROD2 or TARG1 were treated overnight with 100 ng doxycycline/ml, fixed with PFA, stained with primary antibodies (monoclonal antibodies used: MACROD1 (28C11), MACROD2 (18D12), TARG1 (3A5)), visualised using an AlexaFluor488-coupled secondary antibody and analysed with confocal microscopy. **(b)** HeLa Flp-In T-REx control cells were treated and analysed as in panel (a). **(c)** HeLa cells were transfected with the indicated N- or C-terminally GFP-tagged constructs and analysed using live-cell confocal microscopy. **(d)** HeLa cells were transfected with constructs expressing mTurquoise2 targeted to mitochondria and either mRuby2-labeled MACROD1 full-length construct or an N-terminal truncation lacking amino acids 1–77, followed by live-cell confocal imaging. Scale bars represent 10 µM.

We next generated stable HeLa Flp-In T-REx cells as described before^51^, overexpressing the untagged full-length proteins upon induction with doxycycline. In these cell lines, the addition of doxycycline induced specific protein expression as analysed by Western Blot (Supplementary Fig. 2c). We next used these cell lines to determine the intracellular localisation of these three enzymes. MACROD1 was present in defined cytoplasmic structures (Fig. 2a), which could be the mitochondria that MACROD1 was reported to reside in before^32^. We also tested this antibody on cells lacking MACROD1 and observed that the signal disappeared completely (Supplementary Fig. 2d), in contrast to the Abcam antibody which leaves a nuclear staining in knockdown cells^33^. For MACROD2, no specific signal could be detected, as a similar signal was present in control cells (Fig. 2b). TARG1 in this experiment resides preferentially in the nucleus with additional cytoplasmic localisation (Fig. 2a). However, fixation of the cells can influence the apparent localisation of proteins^52^ and therefore we next studied the hydrolases’ localisation using live-cell imaging. We generated both N- and C-terminally GFP-tagged constructs, which appear to be stable and are of the correct size, as analysed by Western Blot (Supplementary Fig. S3a,b). In living cells, the N-terminally tagged MACROD1 localised exclusively to the nuclear compartment where it also appeared to accumulate in nucleoli (Fig. 2c). In contrast, the C-terminally tagged MACROD1 localised to mitochondria. MACROD2 appeared more diffusely in both the cytoplasmic and nuclear compartment (Fig. 2c). Unlike MACROD1, MACROD2 was excluded from nucleoli without notable influence of the N- or C-terminal position of the GFP-tag. TARG1 resided most prominently in nucleoli with additional signals in the nucleoplasm and weaker in the cytoplasm (Fig. 2c), irrespective of the tag placement, comparable to previous findings using live-cell imaging^39^.

We next analysed the subcellular distribution of MACROD1 in more detail, because for MACROD1 contradicting reports have been published reporting different localisations. We co-transfected HeLa cells with a C-terminally mRuby2-tagged MACROD1 plasmid and mTurquoise2 constructs localising to different organelles and structures and analysed their co-localisation using live-cell confocal microscopy (Supplementary Fig. S4a). C-terminally tagged MACROD1 localised to mitochondria but not to any of the other structures studied, such as the nucleus or Golgi apparatus. To exclude any artefacts arising from the GFP-tag or overexpression, we co-stained endogenous MACROD1 and the outer mitochondrial membrane protein TOM20 in RD cells. MACROD1 was readily visible in these cells and co-localised with TOM20 (Supplementary Fig. S4b). These images thus confirm that the observed cytoplasmic structures are mitochondria, in which the overexpressed MACROD1 was located in live-cell imaging. Lastly, we performed cellular fractionation to exclude any artefacts arising during preparation of either fixed or live-cell microscopy samples and observed a MACROD1 signal in the mitochondrial fraction, together with the mitochondrial matrix protein HSP60, but not in the nuclear fraction (Supplementary Fig. S4c). This is in accordance with several mass spectrometry studies, which have identified peptides corresponding to MACROD1 in the mitochondrial matrix^53^. These mass spectrometry studies have identified MACROD1 peptides in their datasets covering most of the protein, however, none have identified peptides belonging to the N-terminus (Supplementary Fig. S4d). This makes it highly likely that the endogenous MACROD1 localises to mitochondria and suggests that upon translocation into mitochondria the N-terminus is cleaved off. This would explain why no peptides mapping to the first 77 amino acids were identified^53^, and also accounts for the differences between MacroD1’s predicted and observed sizes (Supplementary Fig. 3b). Further analysis of the full MACROD1 sequence unveiled two potential mitochondrial targeting sequences (MTS) with cleavage sites at amino acid 35 and 78, respectively, depending on the program used for prediction (Supplementary Fig. S4e). We generated an mRuby2-labeled construct lacking the first 77 amino acids. This abolished mitochondrial localisation in live-cell imaging (Fig. 2d), indicating that this region of the protein is essential for its trafficking to mitochondria. This construct now localised predominantly to the nucleus but not nucleoli and to a lesser degree to the cytoplasm. This implies that MACROD1 might be able to shuttle into the nucleus for example as part of a mitochondrial stress response, as there are other examples of proteins which are partially imported, the N-terminus cleaved and then released to start a nuclear response^54^.

### Loss of MACROD1 and TARG1 disrupts organelle structure

**Figure 3:**
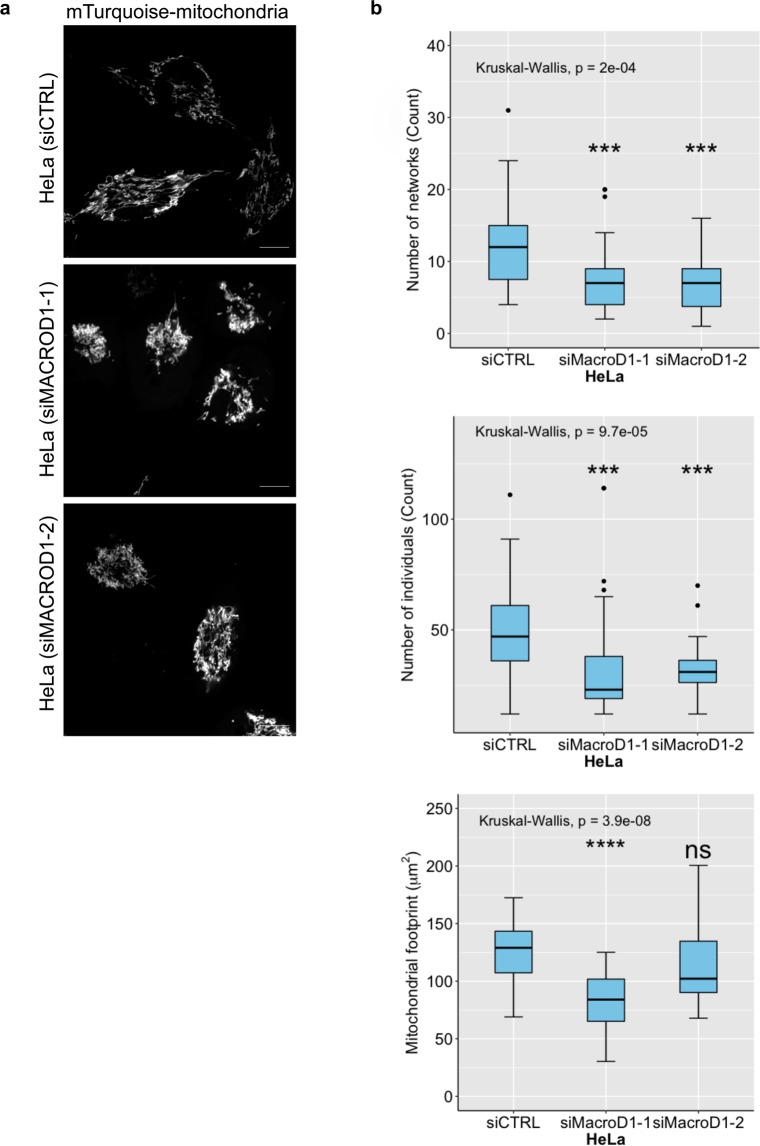
Loss of MACROD1 leads to altered mitochondrial morphology. **(a)** HeLa cells were seeded on Ibidi 8-well slides and transfected with indicated siRNAs and a plasmid containing mTurquoise2 targeted to mitochondria. Cells were analysed live using confocal microscopy. A representative image of 3 independent experiments is shown. **(b)** Automated quantification of at least 50 cells per condition using the mitochondrial network analysis (MiNA) macro tool in ImageJ. Scale bars represent 10 µM. Statistical analysis was performed by a Wilcoxon rank-sum test for comparison of MACROD1 siRNA treated cells with siCTRL treated cells (ns = not statistically significant (P > 0.05), ***P  <  0.001, ****P < 0.0001).

Because we were unable to detect endogenous MACROD2 in most cell lines and could not identify a specific intracellular localisation, which might hint at its participation in defined processes, we focused our further analyses on MACROD1 and TARG1. Using CRISPR/Cas9 we generated *MACROD1* knockout RD cells (Supplementary Fig. 5a) and U2OS *OARD1* knockout cells (Supplementary Fig. 5b). These cells did not show any obvious proliferative defects (data not shown). Because MACROD1 localises to mitochondria, we addressed the fate of these organelles in a *MACROD1*^−/−^ background using confocal microscopy and MitoTracker Red CMXRos. We selected four different clonal lines for these analyses (Clones MD1-A-1, MD1-A-2, MD1-B-1 and MD1-B-2 in Supplementary Fig. 5a). A subtle change in mitochondrial morphology was observed in all four clonal lines, which can be described as mitochondrial fragmentation (Supplementary Fig. 6a). Knocking down *MACROD1* using functional siRNA (Supplementary Fig. 6b) and subsequent staining of mitochondria using a TOM20 antibody for fixed HeLa cells showed a similar effect (Supplementary Fig. 6c). Cells transfected with control siRNA had elongated and interconnected mitochondria, which appeared more fragmented in *MACROD1*^−/−^ cells. Lastly, HeLa cells transfected with siRNA and analysed using live-cell imaging with a co-transfected mitochondrial marker showed a similar phenotype (Fig. 3a). Detailed analysis using automated, unbiased scripts in ImageJ revealed that *MACROD1* knockdown cells had less mitochondria which were less interconnected (Fig. 3b). The mitochondrial footprint, or area, was significantly reduced with one siRNA but not with the other, although a similar trend was visible. This implied that MACROD1 is important for mitochondrial function and that lack of MACROD1 through an unknown process leads to the observed changes in mitochondrial morphology.

**Figure 4:**
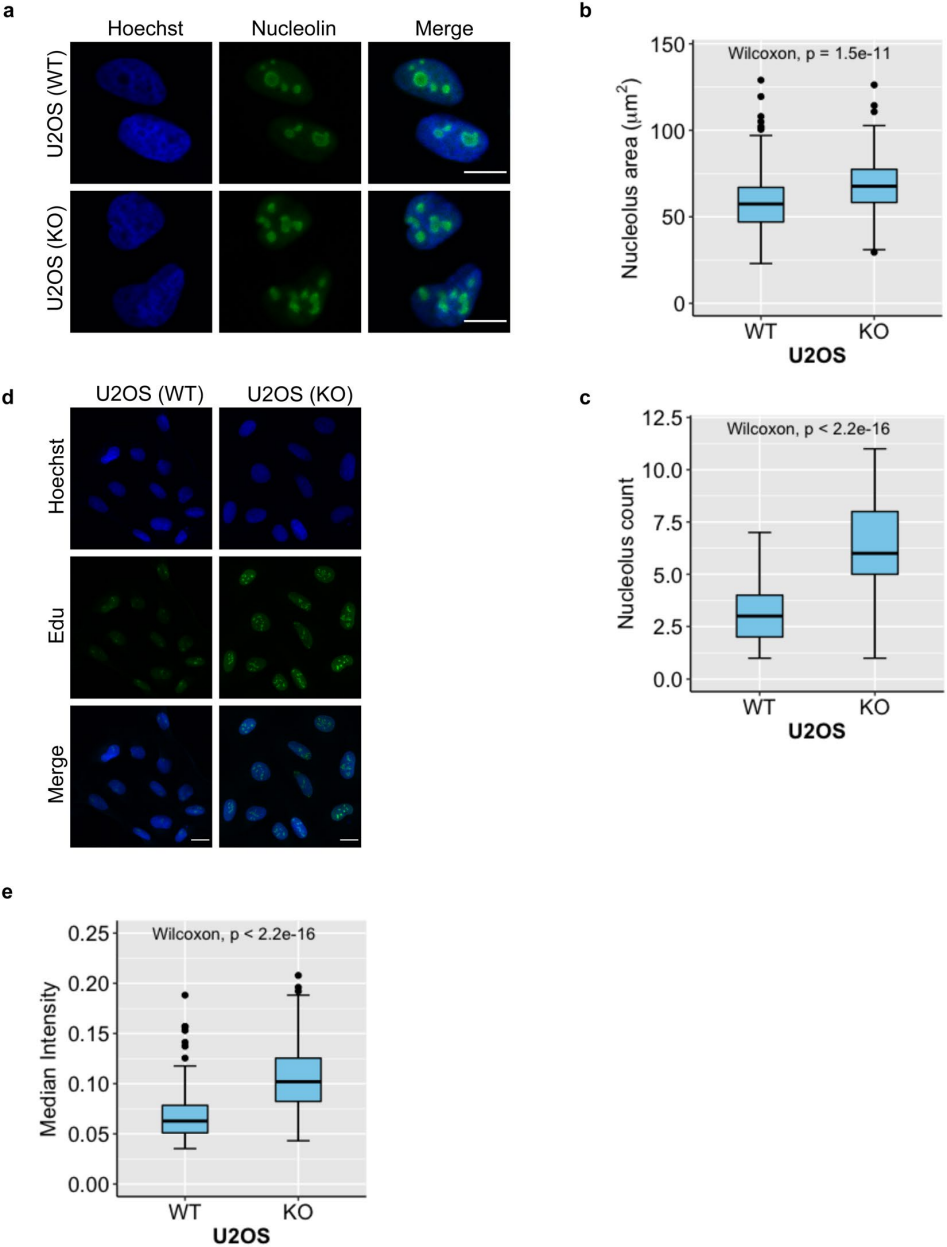
Loss of TARG1 disrupts nucleolar structure and function in U2OS cells. (**a**) *OARD1*^−/−^ and control U2OS cells (KO and WT, respectively) were seeded and fixed in PFA on glass cover slips. A nucleolin antibody was used as to stain nucleoli and was visualised using an AlexaFluor488-coupled secondary antibody. DNA was visualised using Hoechst staining. A representative image of 3 independent experiments is shown. (**b**) and (**c**) Quantification of (a) based on automated analysis using pipelines in CellProfiler for nucleolar area and the number of nucleoli per cell, respectively. (**d**) *OARD1*^−/−^ and control U2OS cells were seeded in 8-well Ibidi slides and fixed in PFA. Transcription rates were measured using a CLICK-iT kit (EdU) and (**e**) quantified as a median intensity per nucleus using CellProfiler software. Scale bars represent 10 µM. At least 200 cells were measured for quantification of nucleolus number/footprint and transcription rate presented in b and e, respectively. Statistical analysis was performed by a Wilcoxon rank-sum test and corresponding p-values are shown in plots.

Likewise, to study the effect of loss of the TARG1 protein, we used CRISPR/Cas9-mediated *OARD1* knockout cells and analysed nucleolar structure visualised by nucleolin staining in fixed samples (Fig. 4a). We chose U2OS cells for analysis of TARG1, as its expression is similar amongst the cell lines tested (Fig. 1) and U2OS have been used in the two earlier studies addressing TARG1 (refs. ^26,39^). A striking difference between wildtype and knockout cells was that the number of nucleoli increased together with the total nucleolar area in *OARD1*^−/−^ cells (Fig. 4b,c). We tested different the knockdown efficiency of multiple siRNAs (Supplementary Fig. 7a) to be able to assess whether this effect was also detectable after siRNA mediated knockdown. For this experiment we used RPE-1 cells because these cells have been immortalized with hTERT and resemble primary cells much closer than U2OS or HeLa cells. Similar to these tumour cells, RPE-1 cells revealed a nucleolar phenotype upon TARG1 knock-down (Supplementary Fig. 7b). As nucleoli are hotspots for rDNA transcription, we next asked whether loss of TARG1 might interfere with this. For this purpose, we generated labelled nascent RNA using a CLICK-iT kit and addressed rRNA synthesis. We found that the *OARD1*^−/−^ cells were transcriptionally more active than their wildtype counterparts when analysed as median intensity per nucleus (Fig. 4d,e). TARG1 thus appears to be essential for nucleolar morphology and function.

### Identification of interacting proteins using BioID

**Figure 5:**
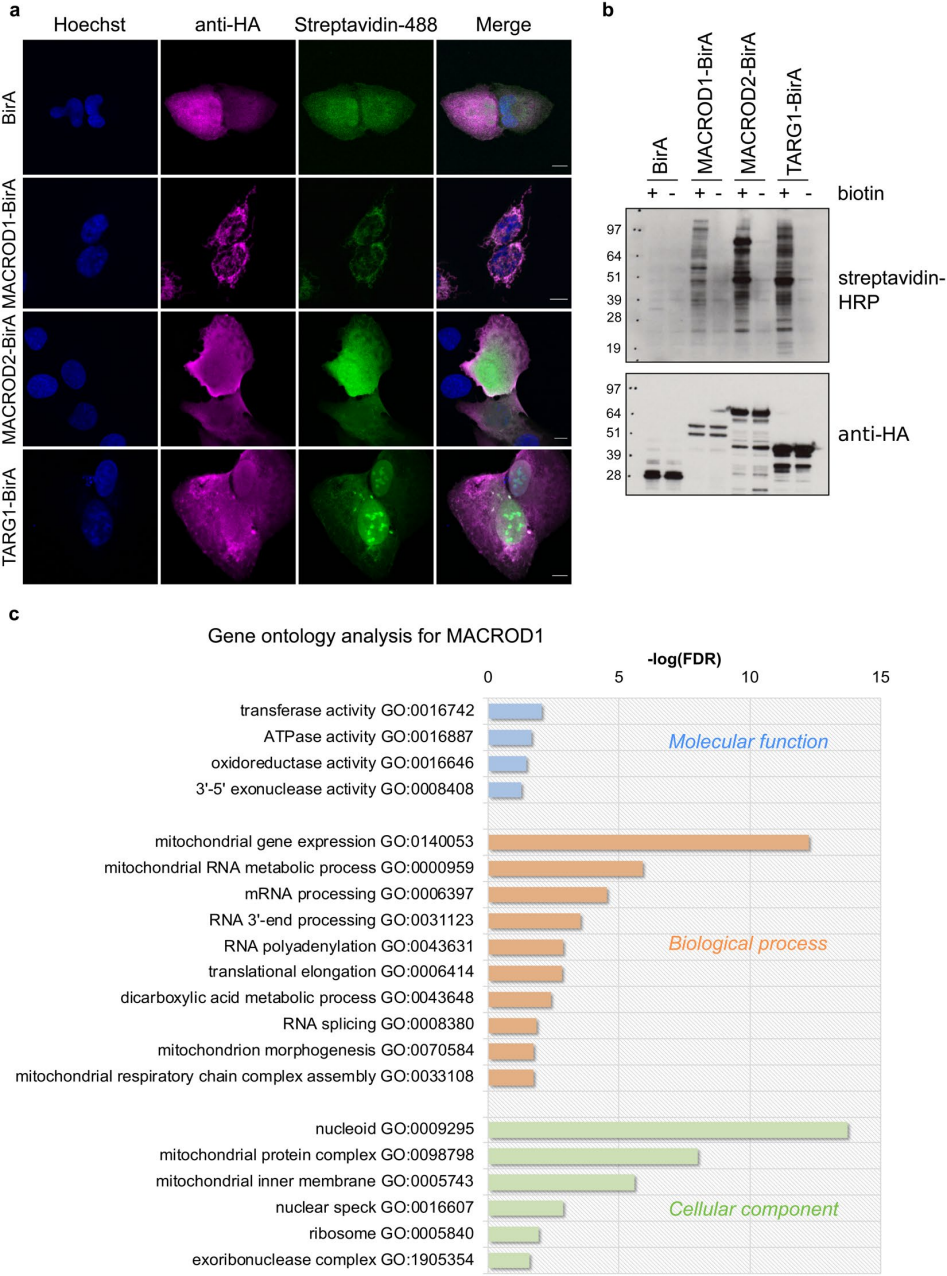
A BioID interaction screen identifies interactors for MACROD1 and TARG1. **(a)** HeLa cells seeded on glass coverslips were transfected with plasmids expressing C-terminally BirA-R118G-fused MACROD1, MACROD2 or TARG1, treated with 150 µM biotin overnight and fixed using 3.7% PFA. The cells were stained using streptavidin-AlexaFluor488 or with an HA-selective antibody and analysed using confocal microscopy. **(b)** HeLa cells were transfected as in (a), treated with 50 µM biotin overnight and lysed in RIPA buffer. Lysates were analysed using Western Blotting and streptavidin-HRP or an HA-selective antibody (whole blots are shown). **(c)** Gene ontology using PANTHER was performed with the proteins identified as interactor for MACROD1 and the enriched cellular components, molecular functions and molecular processes are displayed. Scale bars represent 10 µM. FDR = false discovery rate.

Our observations regarding expression and localisation of MACROD1 and TARG1 as well as the phenotypes of knockout cells provide information of their physiological roles, but do not explain how they function at the molecular level. For TARG1, a tandem affinity purification (TAP)-based interaction screen has been published, where many ribosomal proteins were identified as interactor^39^. Because TARG1 interacts with RNA, it remains unclear which of these ribosomal proteins interact directly or through their associated RNA components in the mild conditions used for a TAP-based interaction screen. To determine their interactors and potential substrates in living cells without having to immunoprecipitate from a cell lysate, we created C-terminal fusions with a promiscuous biotin ligase, BirA-R118G, which biotinylates all proteins in close proximity. This biotin label stays after the interaction dissipates and was developed to identify transient interactions using mass spectrometry^55^. Using this method, we can also visualise where these proteins have been over a longer time period instead of taking snapshots using confocal microscopy. We first fixed the transfected cells and used a streptavidin-AlexaFluor488 conjugate to determine where the biotinylation can be found and in parallel analysed the constructs’ localisation using their HA-tag. The cells were fixed 24 hours after biotin addition, which ought to allow enough time to catch the majority of interactions. The overexpressed BirA-R118G-fusion proteins localised like the untagged proteins studied in fixed HeLa Flp-In T-REx cells (Figs. 2a and 5a). Regarding interactors, for MACROD1 and MACROD2 mediated biotinylation the signal corresponded to the localisation of the MACROD1 and MACROD2 proteins themselves, i.e. in response to MACROD1-BirA the signal was almost exclusively mitochondrial, while for MACROD2-BirA the signal was more diffuse. TARG1-BirA resulted in an enrichment of nucleolar signals, consistent with its primary location in live-cell imaging (Fig. 2b)^39^, despite the fact that upon fixation also the TARG1-BirA fusion protein lost its nucleolar localisation. Together, these findings support the notion that MACROD1 and TARG1 have primary locations in mitochondria and in nucleoli, respectively (Fig. 5a). We also tested the C-terminally tagged BirA constructs on Western Blots and observed that distinct biotinylation patterns appeared for the three hydrolases after overnight incubation with biotin, indicating that they interacted with different proteins (Fig. 5b).

As proteins are labelled in their normal cellular context and then lysates generated under very harsh conditions, which disables any remaining enzymatic activity, this procedure should identify the relevant interactions occurring under state-steady conditions in cells and not ones artificially induced during cell lysis. To determine interaction partners, HEK293T cells were transiently transfected with plasmids expressing BirA only as control and MACROD1 or TARG1 fused to BirA and incubated for 24 hours with biotin to allow labelling of interacting proteins. Biotinylated proteins were then enriched using a streptavidin-matrix and analysed by mass spectrometry after an on-bead digest. We excluded MACROD2 for these experiments, as the MACROD2-BirA fusion protein appears to be unstable (Fig. 5b). We selected all proteins in the MACROD1 and TARG1 experiments enriched >2 fold over background control in two experiments (Supplementary Table S2). The control BirA samples included many carboxylases as was expected from biotin-utilising enzymes. We verified the mass spectrometry results with a pull-down experiment between TARG1 and DHX57, one of the top hits which was predicted to reside in nucleoli, and between MACROD1 and the mitochondrial DNA-directed RNA polymerase POLRMT (Supplementary Fig. 8). Next, we compared the curated list of interactors for both proteins and found a large overlap between the two datasets, which could be explained by a possible interaction between the macrodomain and these interactors through ADP-ribose binding. However, these proteins could also represent artefacts arising due to overexpression of the constructs and were therefore removed from the datasets (Supplementary Table S3). We performed a Gene Ontology (GO) analysis with the curated dataset and found that the top terms for MACROD1 included mainly proteins involved in RNA processing and with a mitochondrial localisation (Fig. 5c). Based on these data and on our localisation studies, we hypothesize that MACROD1’s main function is in mitochondria and more specifically at the mitochondrial nucleoid, possibly in an RNA metabolic pathway that will need to be further defined.

**Figure 6:**
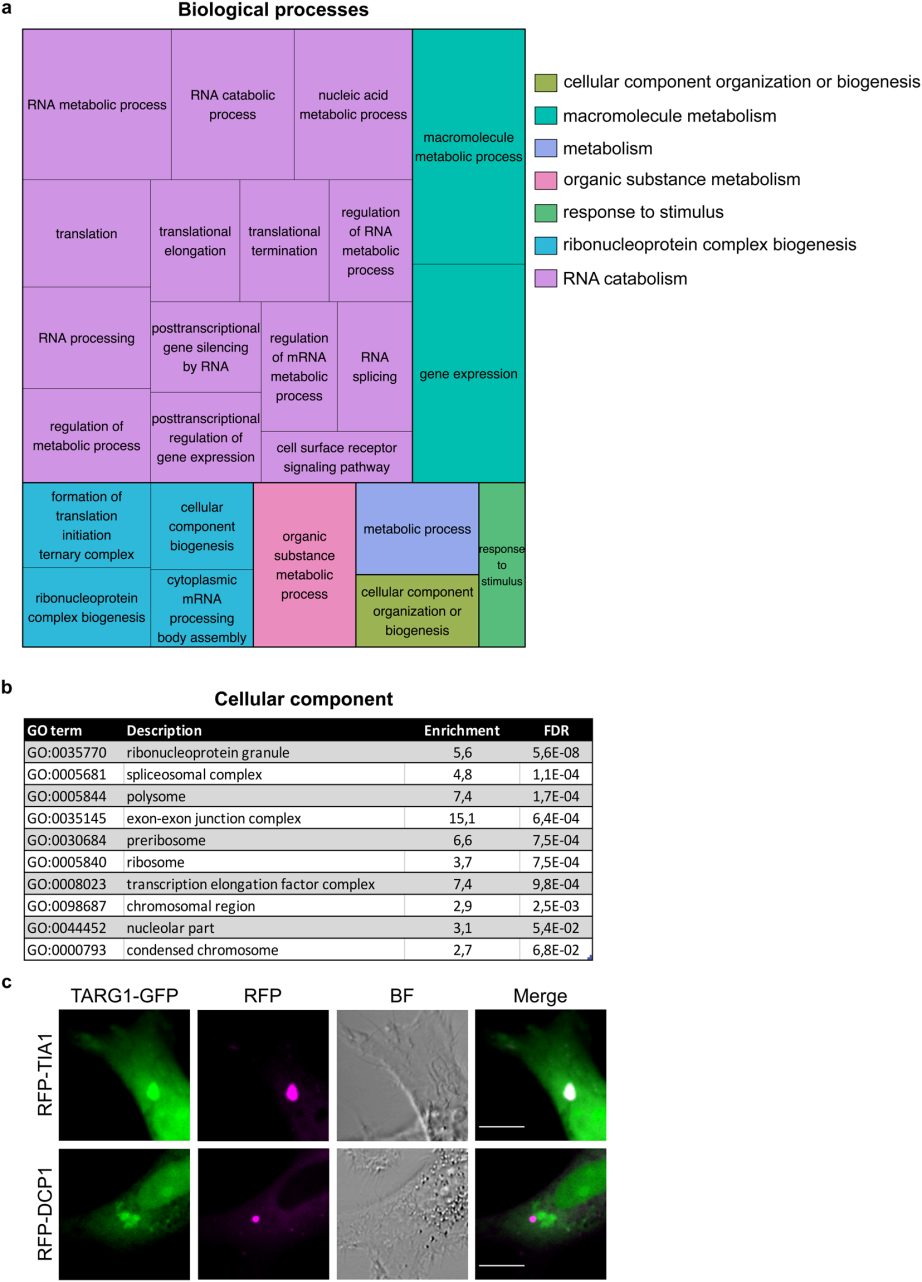
The TARG1 interactome is highly enriched for RNA metabolic processes in diverse cellular compartments. (**a**) A treemap plot was generated with the Gene Ontology terms from the TARG1 BioID analysis highlighting enriched biological processes. The visualization of Gene Ontology functional enrichment for biological processes was made using REVIGO (http://revigo.irb.hr/). A list of the GO-terms is available as Supplementary Table S4. (**b**) List of the most significant cellular component terms associated with the interactors in the TARG1 BioID analysis obtained using the PANTHER toolkit. (**c**) HeLa cells were transfected with plasmids encoding N-GFP-TARG1 and RFP-TIA1 or RFP-Dcp1a, the latter two as markers of stress granules and P-bodies, respectively, and analysed using live-cell confocal microscopy. Scale bars represent 10 µM. BF = brightfield; FDR = false discovery rate; GO = gene ontology; RFP = red fluorescent protein.

For TARG1 we performed the same GO analysis but due to complexity of the results decided to display the findings more graphically (Fig. 6a) and list the GO terms with their corresponding p-values and false discovery rates in a table (Supplementary Table S4). As for MACROD1, the most significantly enriched GO terms were connected to RNA metabolism and to ribosome biogenesis as described before^39^. The cellular component analysis with TARG1 interactors was less clear, as not only nucleoli and nuclear GO-terms were identified as expected, but also for example the more general term ribonucleoprotein granule. This term encompasses not only nuclear granules, but also P-bodies and stress granules (Fig. 6b). When closer investigating existing data of live-cell imaging with overexpressed TARG1, it is possible that the protein may associate with undefined cytoplasmic structures (Fig. 2b), besides its nuclear and nucleolar localisation^26,39^. Based on this, we performed co-transfection experiments with the stress granule marker TIA1 and the P-body marker Dcp1a (Fig. 6c). Under basal conditions not many granules were visible, however, in those cells which displayed TARG1 dots, these structures co-localised with stress granules, but not with P-bodies. This confirmed that BioID is picking up interactors in the different compartments where the proteins reside, even if it only appears to represent a minor part of the total cellular TARG1 pool under basal conditions. That a mono(ADP-ribosyl)hydrolase should be present in stress granules is not surprising, as it was reported before that these granules contain multiple PARPs^56,57^. It remains to be determined under which physiological conditions the endogenous TARG1 is present in these granules. Based on the BioID interactome for TARG1, we postulate that this enzyme partakes in both nucleolar and possibly cytoplasmic RNA regulatory processes, which need to be confirmed in the future.

## Discussion

Although MACROD1, MACROD2 and TARG1 possess comparable biochemical functions, their uniqueness is dictated by their distinct expression pattern and subcellular localisation, explaining why for example a severe neurodegenerative phenotype can result from loss of only one of three enzymes^26^. MACROD1 and TARG1 are rather ubiquitously expressed while MACROD2 expression is most restricted. MACROD1 is specifically enriched in tissues with high energy demand, such as skeletal muscle and heart. Because *MACROD1* knockout mice appear to be viable^33^ as well as our knockout cells, it may be required only when tissues are stressed, by strenuous exercise for example, which would place a burden on mitochondrial capacity in these tissues. Considering the detectable expression of MACROD2 only in cells of neuronal origin, it is likely that MACROD2 has a dedicated role to fulfil there. The ubiquitous TARG1 expression unveils no clues as to its function. Based on these data, we would suggest that for future studies of MACROD2 cells of neuronal origin are probably the best model, such as SH-SY5Y cells, and for MACROD1 skeletal muscle cells, such as RD cells. Based on our findings and a review of the literature, we made a recommendation of the currently available antibodies that are best suited to detect MACROD1, MACROD2 and TARG1 in future studies (Table 2).

**Table 2:** Antibodies recommended for Western Blot and Immunofluorescence.

Application	Antigen	Antibody name	Remarks
**Western Blot**			
	MACROD1	25E9	Shows additional bands; not suitable for IF. Gives a stronger signal than the 28C11 antibody.
	MACROD2	18D12 or HPA049076	The HPA antibody recognizes many larger bands in addition to a band at ± 28 kD; not suitable for IF.
	TARG1	3A5	Shows an additional band in HeLa Flp-In T-REx
**Immunofluorescence**			
	MACROD1	28C11	Slightly weaker staining than 23E9 but highly specific.
	MACROD2		No suitable antibody was found; the polyclonal antibody used before^38^ is not available.
	TARG1	PA5-56043	This antibody shows the most promising localisation in IF but needs validation in WB.

Table 2 summarises the recommended antibodies for Western Blot and immunofluorescence analysis of MACROD1, MACROD2 and TARG1 and describes their limitations.

The three hydrolases localise to different compartments. For MACROD1 in particular the available literature does not reveal a consistent picture because cytoplasmic, nuclear and mitochondrial localisation and functions have been suggested. We confirm that MACROD1 localises to mitochondria dependent on an N-terminal MTS. Upon its removal, the protein relocalises to the cytoplasm and the nucleus. Thus far, mainly nuclear functions have been described for MACROD1, for example in^34,35^, which has multiple possible explanations. One is that the MTS may be masked or cleaved in cells prior to being localized to mitochondria. This might result in cytoplasmic and nuclear localisation, similar to our observation with the N-terminally truncated mutant that no longer is mitochondrial. Alternatively, some of these nuclear interactions may result from the fusion of an N-terminal tag^31,34,58^, which can result in an inaccessible MTS leading to aberrant nuclear localisation, as seen in our live-cell imaging (Fig. 2b). This might mimic the RUNX-MACROD1 fusion protein which has been observed in leukemia^47^ and could thus represent a pathological function. For GFP-labelled TARG1 we observed that it not only localises to the nucleoplasm and nucleoli as seen in previous research^39^, but can also be present in cytoplasmic stress granules.

Further focusing on MACROD1 and TARG1, we find that loss of MACROD1 using both CRISPR/Cas9 and siRNA leads to a fragmented mitochondrial architecture. Likewise, we observe that TARG1 is essential for nucleolar homeostasis, as loss of TARG1 leads to an increase of nucleolar number and mass, in both typical cancer cell lines such as U2OS as well as in the non-transformed cell line RPE-1. The observed phenotypes do not necessarily mean that these proteins are directly involved in maintaining the morphology or formation of these organelles, rather we propose that their loss deregulates certain processes, which are essential and indirectly lead to morphological changes when deregulated. Therefore, we have not further addressed the consequences of these morphological aberrations, but rather focused on determining the underlying cause. To address the molecular mechanism through which loss of MACROD1 and TARG1 disrupts organelle structure, we performed a BioID screen to identify interacting proteins. Striking is that both proteins in their respective compartments appear to fulfil a similar role. Based on GO terms enriched in their interactor sets, both MACROD1 and TARG1 appear closely associated with the processing of RNA. Both nuclear and mitochondrial compartments have transcription and replication machineries as well as RNA regulation systems. If ADP-ribosylation plays a regulatory role therein, it appears that both TARG1 and MACROD1 are present to make this modification reversible in their respective compartments. Thus, despite being located at different places, they might have similar function, i.e. MACROD1 in mitochondrial RNA metabolism and TARG1 in nucleolar and possibly cytoplasmic RNA metabolism. Future work will need to address whether endogenous TARG1 behaves in a similar manner, localising to both nucleoli and stress granules. In light of the recent finding that RNA can be ADP-ribosylated^29^, it will be very interesting to dissect whether modification of protein or RNA is of importance for their function. Based on this work, we suggest that MACROD1 and TARG1 are essential for mitochondrial and nucleolar homeostasis, respectively, possibly through RNA metabolic pathways, which future work will have to address in more detail.

## Methods

### Constructs and cloning

These plasmids were a gift from Dorus Gadella: pmTurquoise2-Mito (Addgene plasmid #36208), pmTurquoise2-ER (Addgene plasmid #36204), pmTurquoise2-Peroxi (Addgene plasmid #36203), pmTurquoise2-Golgi (Addgene plasmid #36205) and pmTurquoise2-H2A (Addgene plasmid #36207)^59^. pcDNA3.1-MCS-BirA-R118G-HA was a gift from Kyle Roux (Addgene plasmid # 36047)^55^. RFP-Dcp1a and RFP-TIA1 were described before^60^. *MACROD1*, *MACROD2* and *OARD1* (encoding TARG1) were amplified from Hela cDNA using primers with flanking attB sites suitable for the Gateway System (Invitrogen) and inserted into pDONR/zeo with a Gateway BP reaction. pcDNA5/FRT/TO-MACROD1, -MACROD2 or -TARG1 were generated by performing a Gateway LR reaction according to the manufacturer’s instructions. GFP-labelled plasmids were generated by LR reactions into pDEST47 and pDEST53. pcDNA3-mRuby2-MACROD1 and pcDNA3-mRuby2-MACROD1d77 were generated using primers with flanking HindIII and Nhel restriction sites for subsequent insertion into pcDNA3-mRuby2 for which the pcDNA5 constructs were used as PCR template. pcDNA3-BirA-R118G constructs with full length MACROD1, MACROD2 and TARG1 were generated using primers with flanking EcoRI and BamHI restriction sites and the pcDNA5 constructs as template.

### Tissue culture and cell transfections

All cell lines were kept at a humidified atmosphere at 37 °C with 5% CO_2_ and were cultivated in low (RD) or high (HeLa, U2OS, RPE-1) glucose DMEM (Sigma) supplemented with 10% heat-inactivated fetal calf serum (Sigma). In addition, 5 µg/ml blasticidin and 100 µg/ml hygromycin B were added to the medium of stable HeLa Flp-In T-REx cell lines.

Dharmacon siRNA smartpools (GE Healthcare) or single siRNAs (GE Healthcare) were transfected using Lipofectamine RNAiMax (Invitrogen) as per manufacturer’s instructions at a final siRNA concentration of 10–50 nM. HeLa and U2OS plasmid transfections were done with calcium phosphate and RD cells were transfected with either PolyFect (Qiagen) according to the manual or calcium phosphate.

### Knockout and overexpression cell lines

HeLa Flp-In T-REx cells were transfected with pcDNA5/FRT/TO, pcDNA5/FRT/TO-MACROD1, pcDNA5/FRT/TO-MACROD2 or pcDNA5/FRT/TO-TARG1 and pOG44 (Invitrogen). The transfected cells were selected using 5 µg/ml blasticidin and 200 µg/ml hygromycin. The cells were kept as polyclonal cell lines.

CRISPR-Cas9-mediated knockout MACROD1 and TARG1 cell lines were generated by transfecting RD, HeLa or U2OS cells with pX459v2 containing *MACROD1 or OARD1* specific gRNAs. Transfected cells were selected with 1 µg/ml puromycin for 24 hours. After selection a serial dilution was made in 96-well plates to obtain clonal cell lines. RD cell survival at a concentration of 1 cell per well was zero and therefore all generated cell lines originated from approximately 5 individual cells instead of being monoclonal. The generated cell lines were analysed using Western Blotting to determine knockout efficiency and lines with remaining MACROD1/TARG1 expression were discarded. *MACROD1* sgRNAs used: 5′ CCATCGTCAACGCCGGTGAGTGG 3′ (MD1–1) and 5′ TGGTGATGTCGCTGCGGAGCAGG 3′ (MD1–2). *OARD1* sgRNA used: 5′ATCAGTGAGGATTGTCGCATGGG 3′. The efficiencies of these sgRNAs were determined using high-resolution melting analysis.

### Antibodies and western blotting

Commercially available primary antibodies were used in a 1:1000 dilution unless stated otherwise: anti-GAPDH (Santa Cruz, sc-32233), anti-GFP 1:2000 (Rockland, 600-301-215), anti-MACROD1, (Abcam 122688), anti-HSP60 (Santa Cruz, SC-1052), anti-TOM20 (Santa Cruz, FL-145), anti-HA (3F10, Roche), streptavidin-HRP 1:40.000 (Abcam ab7403), streptavidin-AlexaFluor488 (Dianova), anti-tubulin (Santa Cruz sc23948), anti-nucleolin 1:5000 (Abcam ab13541), anti-POLRMT (Sigma HPA006366), anti-DHX57 1:500 (Abcam ab86784). Monoclonal antibodies recognising TARG1 were raised against the full-length His-tagged protein in rat^39^, against the full-length His-tagged MACROD2 in rat and against His-tagged MACROD1 lacking the N-terminus in mice. The best antibodies were selected using recombinant protein in dot-blot assays and validated on cell lysates. Experiments in this work were performed with hybridoma culture supernatants from clones TARG1 3A5 (rat IgG2b/k), MACROD2 18D12 (rat IgG2b/k), and MACROD1 25E9 or 28C11 (mouse IgG2c/k) at a 1:250 dilution for Western Blot and undiluted for immunofluorescence.

Cell protein extractions were performed using RIPA buffer (150 mM NaCl, 1% Triton X-100, 0.5% sodium deoxycholate, 0.1% SDS, 50 mM Tris-HCl (pH 8.0)) supplemented with protease inhibitor cocktail, followed by sonication on ice or benzonase treatment (Sigma). Proteins were separated on 10–15% gels and blotted onto nitrocellulose. Membranes were blocked with 5% non-fat milk in TBST (TBS from Sigma with 0.05% Tween) for 1 hr at RT, primary antibodies were diluted in TBST and incubated overnight at 4 °C, secondary antibodies were diluted in 5% non-fat milk in TBST and incubated for 1 hr at RT. Blocking and antibody incubations were performed in 2.5% BSA in TBST for streptavidin-HRP analysis. Wash steps were performed in between and after antibody incubations with TBST at RT for at least 5 mins. Chemiluminescent signals were detected by either exposure to film or using the Azure600.

### Cellular fractionation

Cells were trypsinized, washed with sucrose buffer (250 mM sucrose, 2 mM HEPES, 0.1 mM EGTA, pH 7.4), centrifuged and resuspended in this buffer. Cells were homogenized using approximately 25 strokes on a dounce homogenizer. Homogenised cells were centrifuged at 600 × g for 10 minutes at 4 °C. The resulting pellet, containing the nuclei and unbroken cells, was resuspended and homogenised again, after which supernatants were pooled. The resulting pellet was treated with 0.1% Triton-X100 and centrifuged again to clean up the nuclear fraction. The pooled supernatants were centrifuged at 11.000 × g to collect the mitochondria, which were also treated with 0.1% Triton-X100 to obtain clean mitochondrial fractions.

### RT-qPCR

*MACROD1* and *OARD1* mRNA were analysed on a human tissue RNA library (Clontech: Human Total RNA Master Panel II). Reverse transcription was performed on 1 µg RNA using the QuantiTect Reverse Transcription Kit (QIAGEN) according to manufacturer’s instructions. mRNA levels were determined using quantitative real-time PCR with SYBR-Green (QIAGEN) and QuantiTect Primer Assays for *MACROD1*, *OARD1* and *GUS* (QIAGEN Hs_MACROD1_1_SG, Hs_OARD1_1_SG and Hs_GUSB_1_SG, respectively).

### Microscopy

HeLa Flp-In T-REx-MACROD1, HeLa Flp-In T-REx-MACROD2, HeLa Flp-In T-REx-TARG1 and HeLa Flp-In T-REx control cells were seeded onto glass coverslips in 24 well plates. Once adhered, 100 ng/ml doxycycline was added to each well and incubated at 37 °C with 5% CO2 for 12 hours to induce expression. Cell were washed twice with PBS and fixed with 4% paraformaldehyde in PBS. Blocking was done in PBS supplemented with 1% BSA for 1 hour at RT. Primary and secondary antibodies were applied for 1 hour and Hoechst was applied for 5 minutes at RT. Primary antibodies used were undiluted hybridoma culture supernatants: MACROD1 28C11 mouse monoclonal, MACROD2 18D12 rat monoclonal and TARG1 3A5 rat monoclonal. Secondary antibodies used: AlexaFluor594 goat anti-mouse and AlexaFluor594 goat anti-rat (both ThermoFisher) used 1:2000. The same procedure was followed for staining of endogenous MACROD1 in RD cells. For the analysis of mitochondrial morphology upon siRNA transfection cells were transfected using Lipofectamine RNAiMax according to the manual for 48 hours prior to fixation. For quantification of the number and size of nucleoli in *OARD1* knockout cells, cells were fixed in PFA (4%) and stained for nucleolin. Primary antibodies used: MACROD1 28C11 mouse monoclonal undiluted, and TOM20 rabbit polyclonal 1:5000 (Santa Cruz). Secondary antibodies used: AlexaFluor488 goat anti-rabbit and AlexaFluor594 goat anti-mouse (both ThermoFisher) used 1:2000. The same protocol was followed for nucleolin stainings in *OARD1*^−/−^ cells. The nucleolin antibody (Abcam13541) was used in a 1:1000 dilution.

For live-cell imaging of organelle markers and MACROD1 truncations, cells were seeded in glass-bottomed 24-well plates and transfected using PolyFect (Qiagen). After 24 hours the cells were washed in warm PBS and the cells were analysed after 48 hours. The images were taken with the 100x oil immersion objective of the Olympus Fluoview FV1000 confocal microscope.

For the analysis of interactors cells were transfected with BirA constructs, washed in HEPES buffer the next day, supplied with 50 µM biotin (Sigma) overnight and fixed using 4% paraformaldehyde in PBS 48 hours after transfection. The BirA fusion proteins were visualised using an anti-HA antibody in a 1:250 dilution (3F10, Roche); biotinylation was visualised using streptavidin-AlexaFluor488 in a 1:1000 dilution (Dianova). Secondary antibodies used: AlexaFluor594 goat anti-rat (ThermoFisher). All coverslips were mounted on microscopy slides using Prolong Anti-Fade Diamond Mountant (ThermoFisher).

For live-cell imaging of N-and C-terminally labelled constructs cells were seeded in 8-well slides (Ibidi) and transfected using calcium phosphate. After 24 hours the cells were washed in warm HEPES buffer and the cells were analysed after 48 hours. The samples were analysed with the Zeiss LSM 710 Confocal Laser Scanning Microscope equipped with an AxioCam (Zeiss) and a C-Apochromat 40x water immersion objective. For live-cell imaging of mitochondria, cells were seeded in 8-well slides (Ibidi) and transfected with siRNA using Lipofectamine RNAiMax (Invitrogen) according to instructions. After 48 hours, cells were incubated with 50 nM MitoTracker Red CMXRos (Invitrogen) and analysed using a Zeiss LSM 710 as above.

#### CLICK-IT

Wildtype and knockout *OARD1* cells were seeded in 8-well chambers (Ibidi). Nascent transcription was measured using the Click-iT EdU Alexa Fluor 488 Imaging Kit (ThermoFischer) exactly according to manufacturer’s instructions. Images were taken using the Zeiss LSM 710 as above.

### BioID

BioID was performed essentially as described before^55^. HEK293T cells were transfected with either control or C-terminally tagged hydrolase-BirA constructs, washed in PBS after 6 to 8 hours and further cultured in media with 50 µM biotin. 24 hours after addition of biotin, cells were washed once in PBS and frozen at −80 °C or immediately processed by adding RIPA lysis buffer. Samples were collected and diluted with 10 mM Tris (pH 8), to allow efficient benzonase-mediated digestion of DNA for 1–2 hours at 4 °C. Lysates were cleared by centrifugation and incubated with streptavidin-agarose. All subsequent washing steps were performed exactly as detailed before^55^. A tryptic digest was performed on bead using an established FASP protocol^61^. Mass spectrometry was carried out essentially as described previously, using a Q Exactive mass spectrometer (Thermo, Hemel Hempstead) coupled to a Dionex Ultimate 3000 RSLCnano system as described before^62^. Mass spectra were searched against the Human complete proteome database using the Andromeda algorithm through MaxQuant (version 1.5.2.8) or using SINQ^63^. From the resulting list of interactors identified in two experiments, all proteins were removed which were enriched less than 2 times over control. GO term enrichment was performed using the PANTHER toolkit^64^. Significantly enriched GO terms (p < 0.05) were condensed and visualized using ReviGO^65^ and the R package treemap^66^. The list of interactors resulting from this analysis is available as Supplementary Table S2 and raw data are available online.

### Statistical analysis

Mitochondrial morphology was analysed using the mitochondrial network analysis (MiNA) ImageJ macro tool^67^. At least 50 cells were analysed per condition. P values were calculated using two-tailed Kruskal-Wallis test followed by Wilcoxon rank-sum tests for comparisons of siRNA treated cells with control cells (ns = not statistically significant (P > 0.05), *P < 0.05, **P  <  0.01, ***P  <  0.001, ****P < 0.0001). Statistical analysis was performed using R software^68^.

The signal intensity within the nucleus resulting from the Click-iT analysis was quantified using CellProfiler software^69^. The number of nucleoli and total footprint per nucleus were quantified in at least 200 cells per condition using CellProfiler and analysed in R software using a Wilcoxon rank-sum test. The data were visualised using the R-package ggplot2^70^. In box plot graphs, boxes represent the 25–75 percentile range with median and whiskers represent the 5–95 percentile range. Data points outside this range are shown individually.

## Supplementary information

Supplementary information.

Supplementary information2.

Supplementary information3.

Supplementary information4.

## Data Availability

All data generated or analysed during this study are included in this published article (and its Supplementary Information files). Generated reagents such as antibodies, plasmids and stable cell lines are available from the corresponding authors on reasonable request.
