# Supplementary material for: Comparative analysis of MACROD1, MACROD2 and TARG1 expression, localisation and interactome
Source: Sci Rep. 2020 May 19;10:8286. doi: 10.1038/s41598-020-64623-y (PMC7237415; doi:10.1038/s41598-020-64623-y)
Supplement: Supplementary file 1 — Supplementary information. [file 41598_2020_64623_MOESM1_ESM.pdf]

Supplementary Information

Comparative analysis of MACROD1, MACROD2 and TARG1 expression, localisation and interactome

R. Žaja[1*], G. Aydin[1], B. Lippok[1], R. Feederle[2], B. Lüscher[1] and K.L.H. Feijs[1*]

[1] Institute of Biochemistry and Molecular Biology, RWTH Aachen University, Pauwelsstrasse 30, 52074, Aachen, Germany

[2] Monoclonal Antibody Core Facility, Institute for Diabetes and Obesity, Helmholtz Zentrum München, German Research Center for Environmental Health, Neuherberg, Germany

[*] Correspondence should be addressed to R. Zaja (rzaja@ukaachen.de) and K.L.H. Feijs (kfeijs@ukaachen.de)

Supplementary Figure 1 MacroD1, MacroD2 and TARG1 expression according to GTex

a

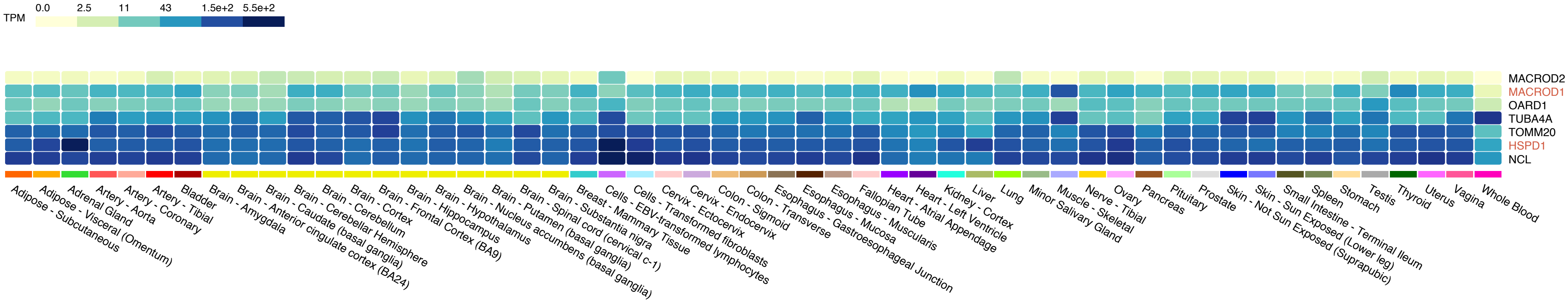

b

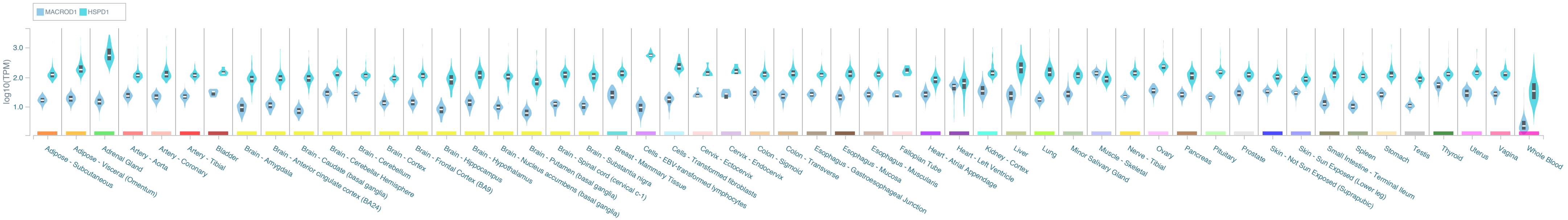

Supplementary Figure 1 *MACROD1*, *MACROD2* and *OARD1* RNA expression in the online GTex (**a**) The RNA expression of *MACROD1*, *MACROD2* and *OARD1* in a set of human tissues was compared, as well as a number of genes encoding for proteins localising to similar intracellular locations. *OARD1* = TARG1, *TUBA4A* = tubulin alpha 4A, *TOMM20* = TOM20, *HSPD1* = HSP60, *NCL* = nucleolin (**b**) The RNA expression of *MACROD1* and *HSPD1* are compared.

The data used for the analyses described in this manuscript were obtained from the GTEx Portal on 08/15/19

Supplementary Figure 2 MacroD1 antibody and HeLa Flp-In T-REx cell line tests

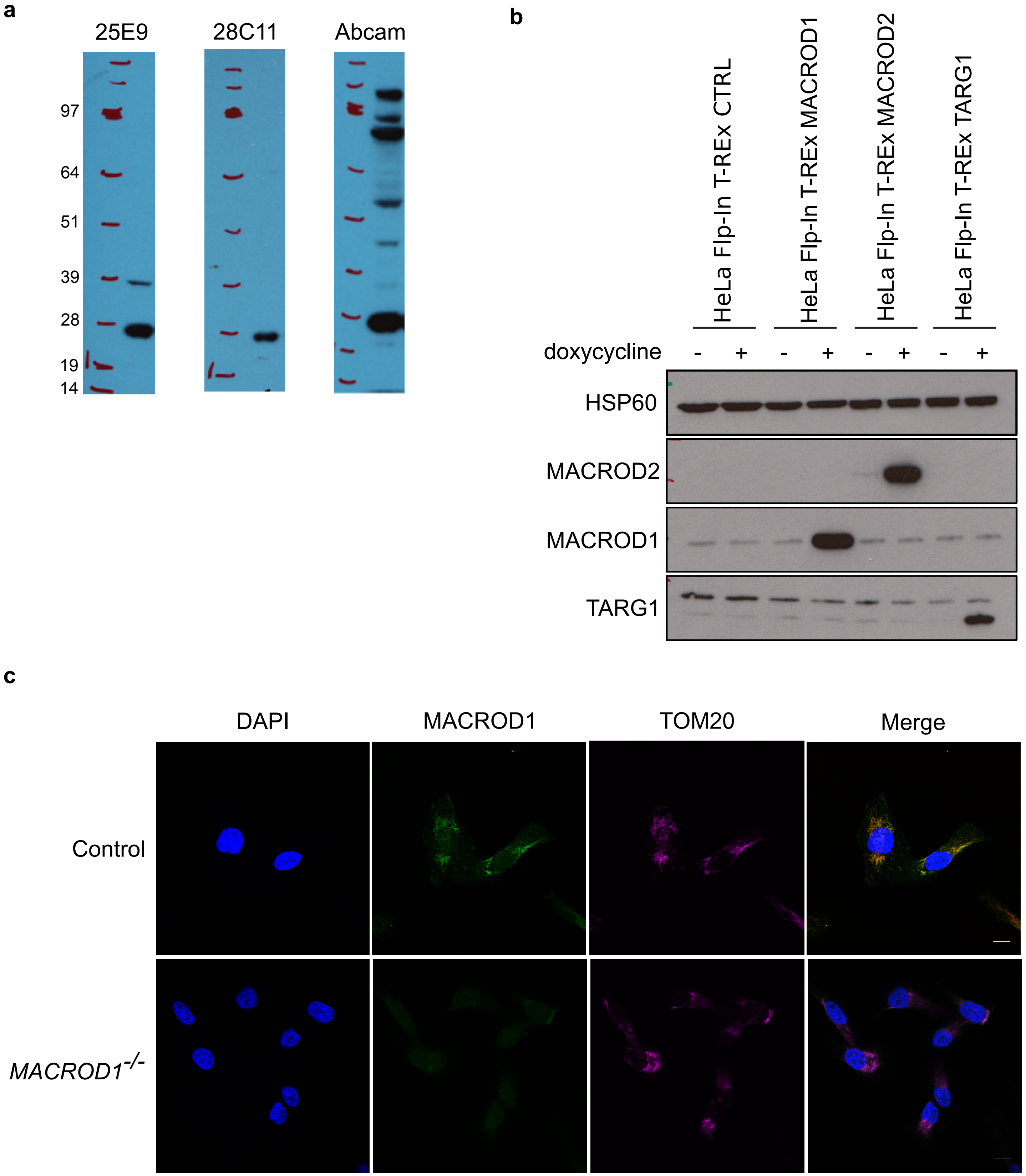

Supplementary Figure 2 MACROD1 antibody and HeLa Flp-In T-Rex cell line tests
(a) RIPA lysates were generated from HEK293 cells and were analysed using Western Blot. Monoclonal antibodies for MACROD1 used: 25E9 and 28C11 and the polyclonal Abcam 122688. **(b)** Indicated HeLa Flp-In T-REx cells were treated overnight with 100 ng doxycycline/ml to induce protein expression, lysed and analysed by Western Blotting. Antibodies used: MACROD1 (25E9), MACROD2 (18D12), TARG1 (3A5) and HSP60. Multiple exposures of the uncropped blots are available in Supplementary Figure 9. **(c)** RD control or *MACROD1* knockout cells were fixed and stained with the monoclonal MACROD1 antibody 28C11 and with a polyclonal TOM20 antibody. Stainings were visualised with AlexaFluor488 and AlexaFluor633.
Scale bars represent 10μM.

Supplementary Figure 3 Analysis of GFP-fusion proteins

a

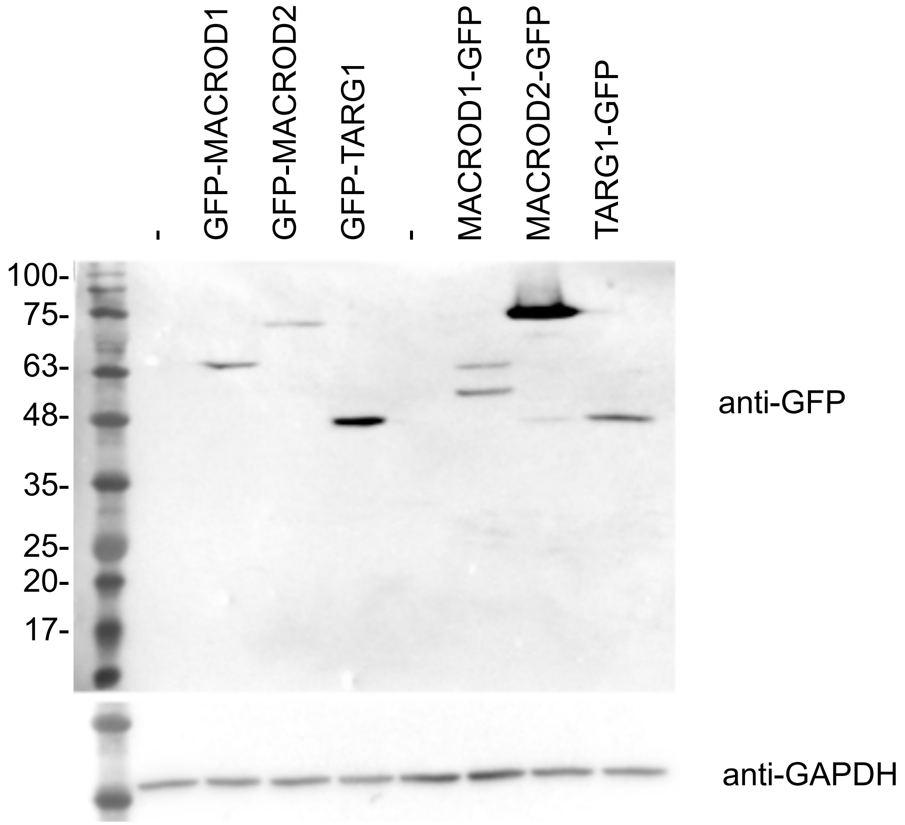

b

	Untagged	with GFP-tag
MACROD1	35.5 kDa (±29 kDa)	62.5 kDa (±56 kDa)
MACROD2	47 kDa	74 kDa
TARG1	17 kDa	44 kDa

Supplementary Figure 3 Analysis of GFP-fusion proteins

(a) N- and C-terminal GFP-tagged MACROD1, MACROD2 and TARG1 were overexpressed in HeLa cells. Lysates were generated in RIPA 36 hours after transfection and analysed using Western Blot with GFP and GAPDH antibodies. The whole blot was first detected with the GFP antibody and subsequently with a GAPDH antibody. **(b)** The predicted molecular weight of the untagged fusion proteins and of the GFP-labelled proteins; the numbers in brackets are the observed molecular weight for MacroD1 when analysing untagged or C-terminally labelled protein.

Supplementary Figure 4 MACROD1 localises to mitochondria

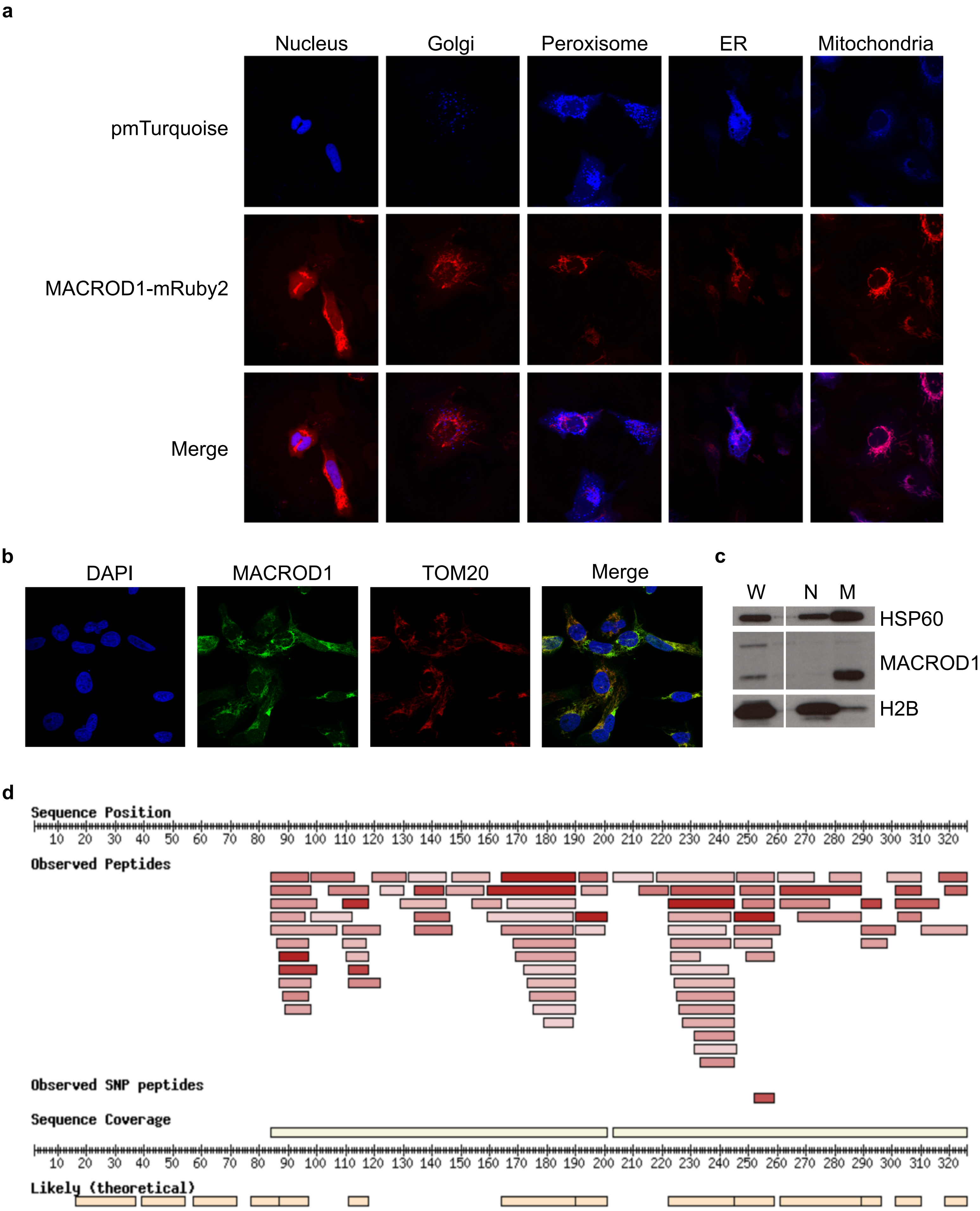

e

Prediction software	Predicted cleavage site	Probability of mitochondrial localisation
MitoFates	Amino acid 37	0.960
MitoProt II v1.101	Amino acid 78	0.998

Supplementary Figure 4 MACROD1 localises to mitochondria

(a) HeLa cells were transfected with plasmids containing mTurquoise2 organelle markers and mRuby2-labeled MACROD1 and analysed by live-cell confocal imaging. ER = endoplasmatic reticulum. **(b)** RD cells seeded on glass coverslips were fixed with 3.7% PFA, stained using MACROD1 and TOM20 antibodies and analysed by confocal microscopy. **(c)** HeLa cells were fractionated and MACROD1 localisation analysed by Western Blot using the 25E9 antibody. The blot was cut in three strips to be able to detect markers for the different fractions and MACROD1 at the same time. HSP60 was used as marker for the mitochondrial fraction, H2B as marker for the nuclear fraction. W = whole cell lysate, N = nuclear fraction, M = mitochondrial fraction. Multiple exposures of the uncropped whole blots are available in Supplementary Figure 9. **(d)** Overview of MACROD1 peptides identified in mass spec datasets (red, with darker colouring for more abundant peptides), the coverage achieved (white) and the theoretical likely to occur peptides (orange) as predicted by PeptideAtlas. **(e)** MACROD1's amino acid sequence was analysed using two different programs, which predicted two different putative mitochondrial targeting sequences and cleavage sites as indicated.

Supplementary Figure 5 CRISPR/Cas9-mediated MACROD1 and TARG1 knockout cells

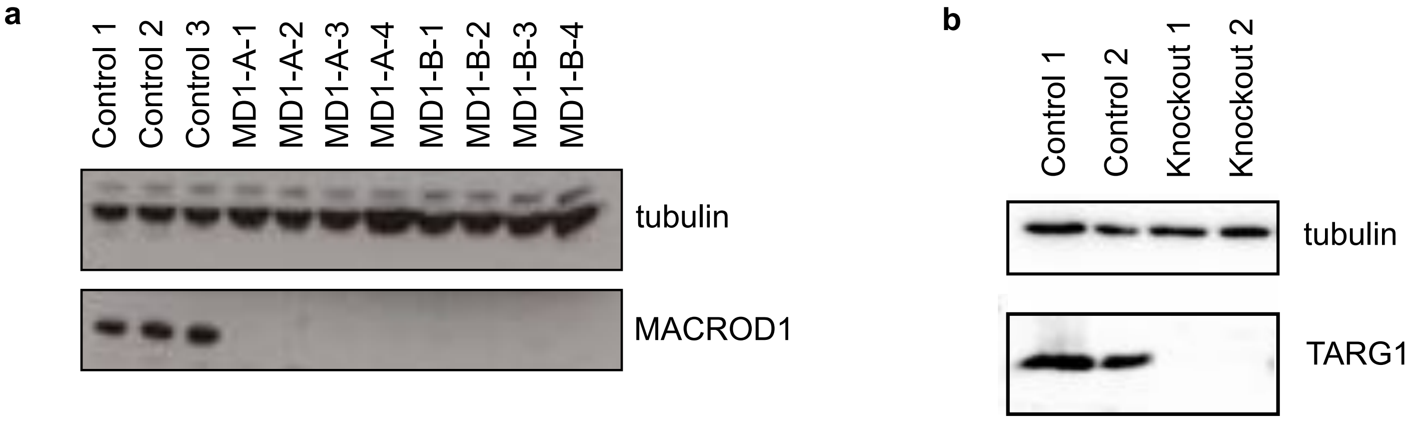

Supplementary Figure 5 Generation of *MACROD1* and *OARD1* knockout cells

(**a**) Stable RD cell lines were generated after transfection with CRISPR/Cas9 constructs and puromycin selection. Loss of MACROD1 was analysed using Western Blot with the 25E9 antibody and tubulin as loading control. The blot was cut to incubate both antibodies simultaneously; two different exposures are available in Supplementary Figure 9. (**b**) Stable U2OS cell lines were generated after transfection with CRISPR/Cas9 constructs and puromycin selection. Loss of TARG1 was analysed using Western Blot with the 3A5 antibody and tubulin as loading control. The blot was cut to incubate both antibodies simultaneously.

Supplementary Figure 6 Loss of MACROD1 leads to a mitochondrial phenotype

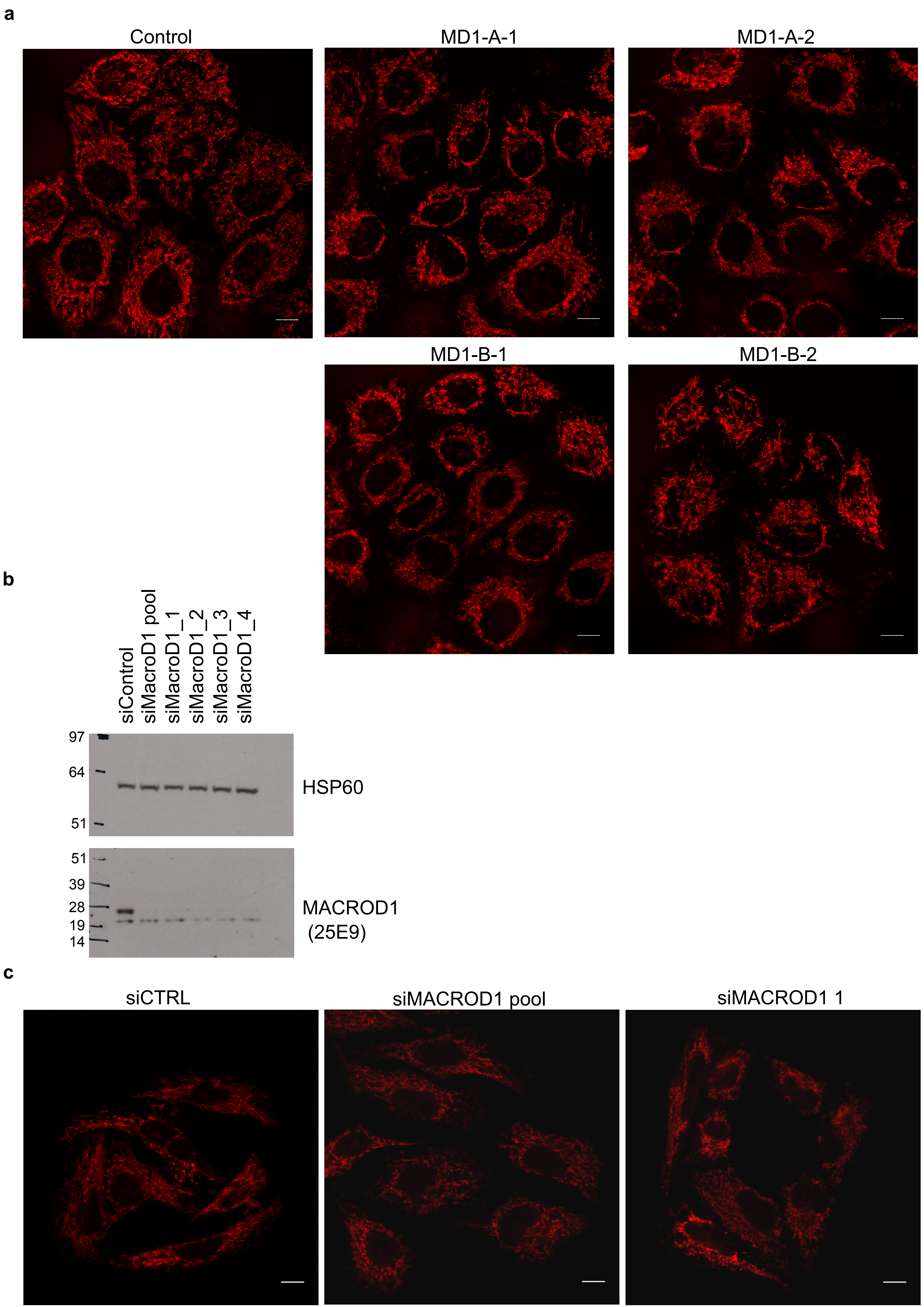

Supplementary Figure 6 Loss of MACROD1 leads to mitochondrial fragmentation

(**a**) RD knockout or control cells were seeded in glass-bottom plates and next day incubated with MitoTracker CMXRos^{TM} for 30 minutes. Cells were analysed live using confocal microscopy. A representative experiment is shown. **(b)** HeLa cells were transfected with a control siRNA or different siRNAs against *MACROD1* for 48 hours, lysed in RIPA buffer and analysed by Western Blot using the monoclonal MACROD1 antibody 25E9 and an HSP60 antibody. (**c**) HeLa cells were seeded on glass coverslips and transfected with siCTRL or siMACROD1 and fixed in PFA 48 hours later, followed by staining with a TOM20 primary antibody and AlexaFluor secondary.

Scale bars represent 10 μM.

Supplementary Figure 7
TARG1 knockdown leads to a nucleolar phenotype in RPE-1 cells

a

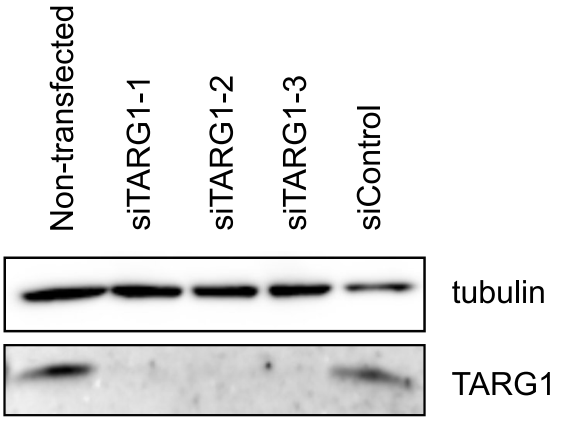

b

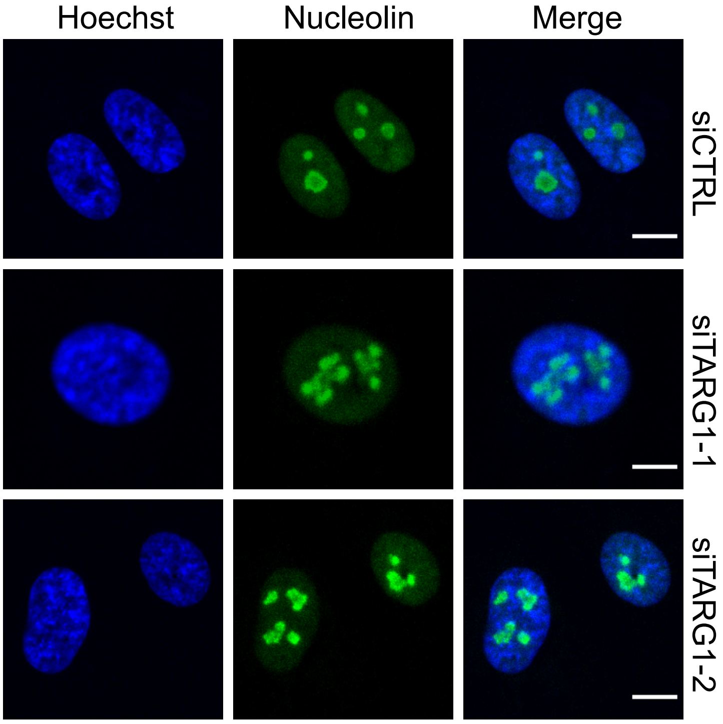

Supplementary Figure 7 Knockdown of TARG1 leads to a nucleolar phenotype in RPE-1 cells
(**a**) U2OS cells were transfected with control siRNA or siRNA against TARG1, lysed 48 hours later and analysed using Western Blot with the 3A5 antibody for TARG1 and tubulin as loading control. The blot was cut to incubate both antibodies simultaneously; different exposures of the uncropped blot are available in Supplementary Figure 9. (**b**) RPE-1 cells were seeded on glass coverslips and transfected with siTARG1 or siControl. 48 hours after transfection, cells were fixed in PFA and stained for nucleolin.
Scale bars represent 10 μM.

Supplementary Figure 8: confirmation of BirA pull-down

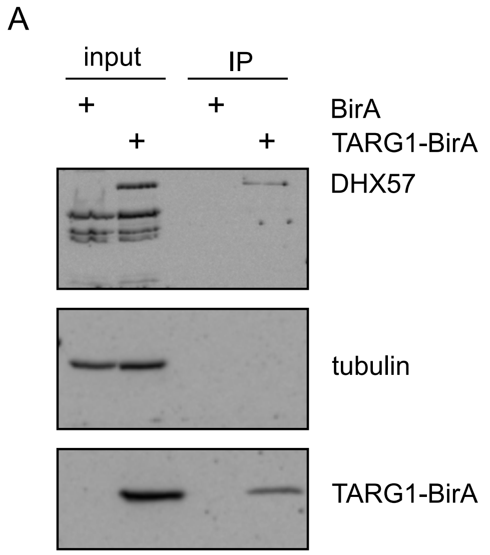

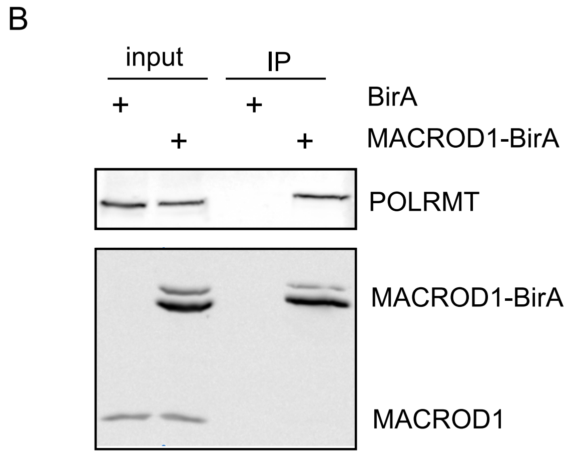

Supplementary Figure 8 BioID interactions between TARG1 & DHX57 and MACROD1 & POLRMT

(**a**) HEK293T cells were transfected with plasmids containing TARG1-BirA or BirA alone and treated with biotin for 24 hours. Lysates were generated and biotinylated proteins enriched using streptavidin-dynabeads. Whole cell lysates and the pull-down were analysed using Western Blot, the blot was cut in three strips and all proteins detected simultaneously. Antibodies used: DHX57, tubulin and TARG1 (3A5). (**b**) HEK293T cells were transfected with MACROD1-BirA or BirA alone and treated with biotin for 24 hours. Lysates were generated and biotinylated proteins enriched using streptavidin-dynabeads. Whole cell lysates and the pull-down were analysed using Western Blot, the blot was cut in two strips which were detected simultaneously, the MACROD1 strip was incubated with a higher sensitivity ECL. Antibodies used: POLRMT and MACROD1 (25E9).

PD = pull-down

Supplementary Figure 9 Full blots
Belonging to Figure 1c

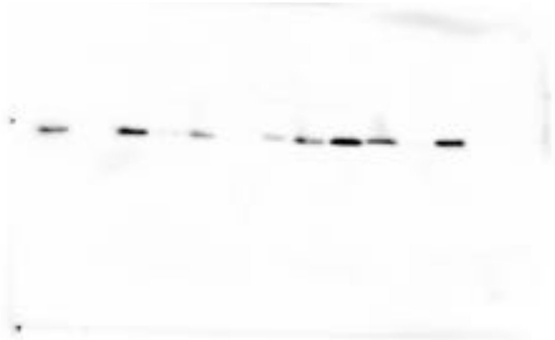

MACROD1

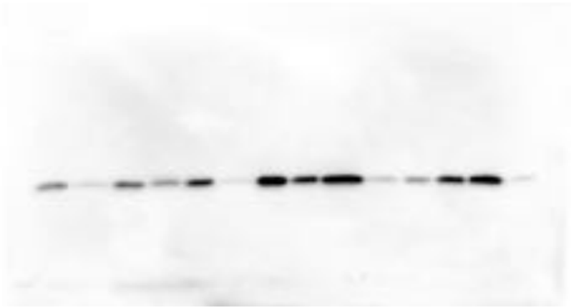

TARG1

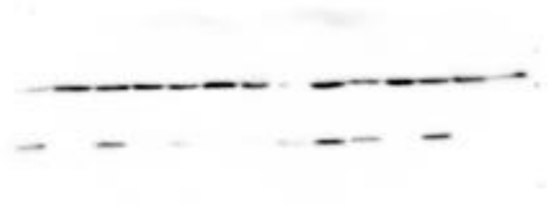

actin (MACROD1 blot)

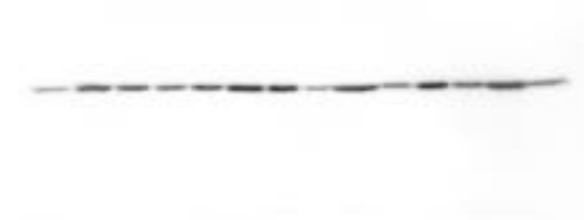

actin (TARG1 blot)

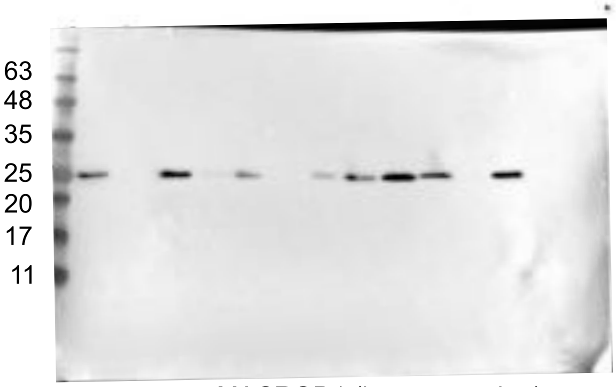

MACROD1 (impose marker)

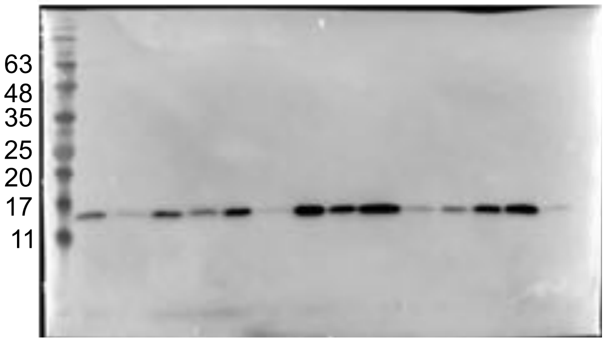

TARG1 (impose marker)

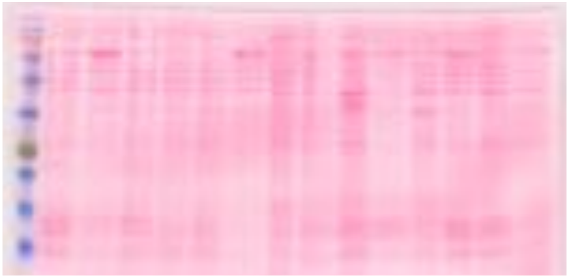

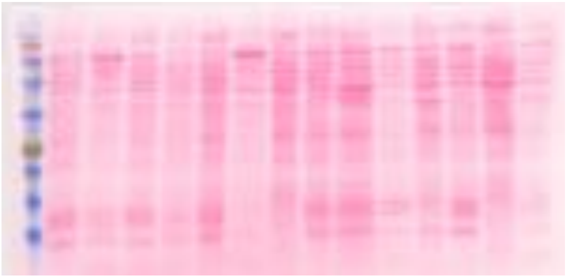

Supplementary Figure 9 Full blots
Belonging to Figure 1d

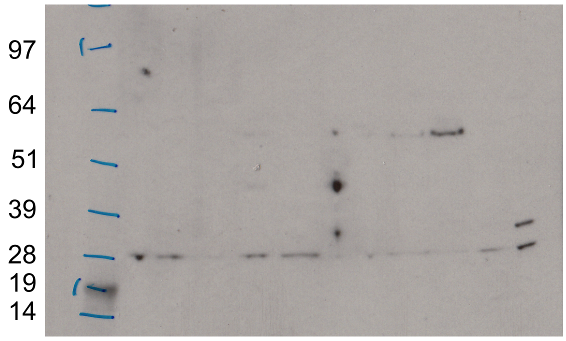

MacroD2

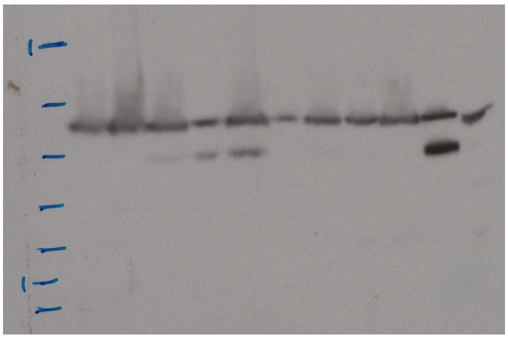

HSP60 (MacroD2 blot)

Supplementary Figure 9 Full blots
Belonging to Supplementary Figure S2b

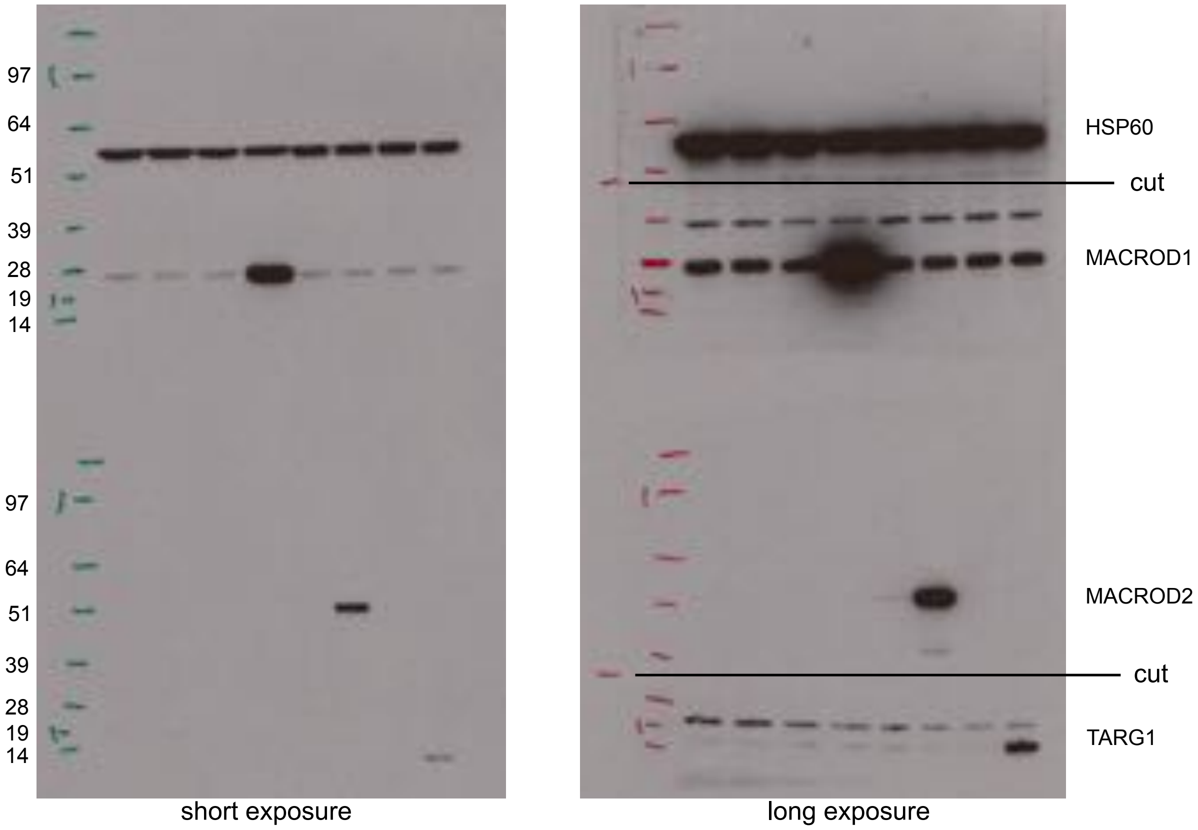

Supplementary Figure 9 Full blots
Belonging to Supplementary Figure S4c

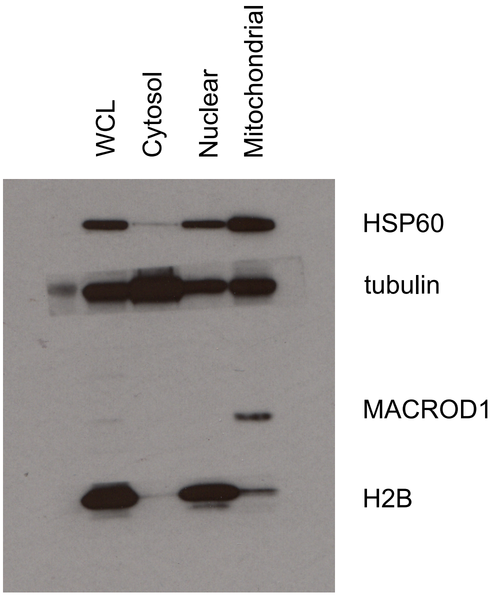

short exposure

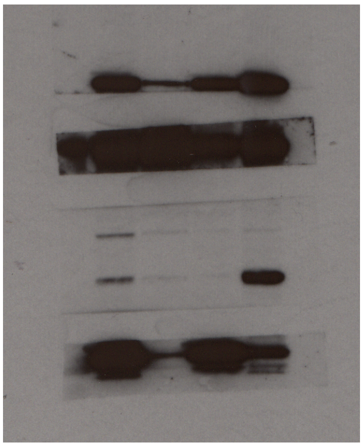

long exposure

Supplementary Figure 9 Full blots
Belonging to Supplementary Figure S5a and S5b

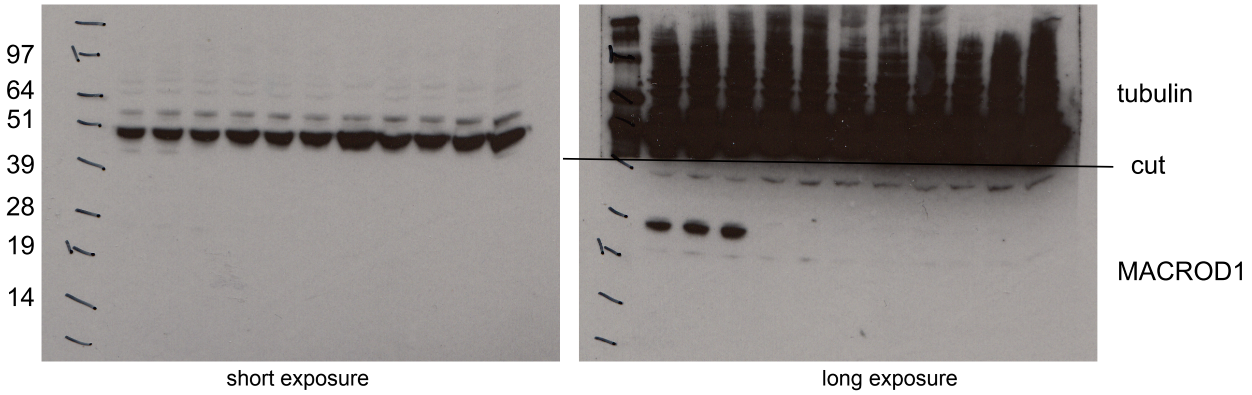

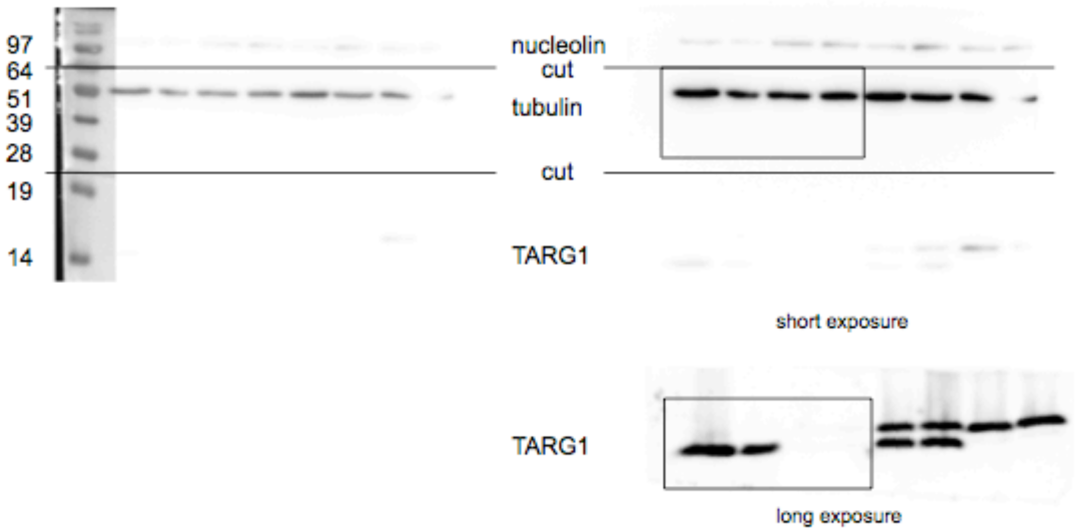

Supplementary Figure 9 Full blots
Belonging to Supplementary Figure S7a

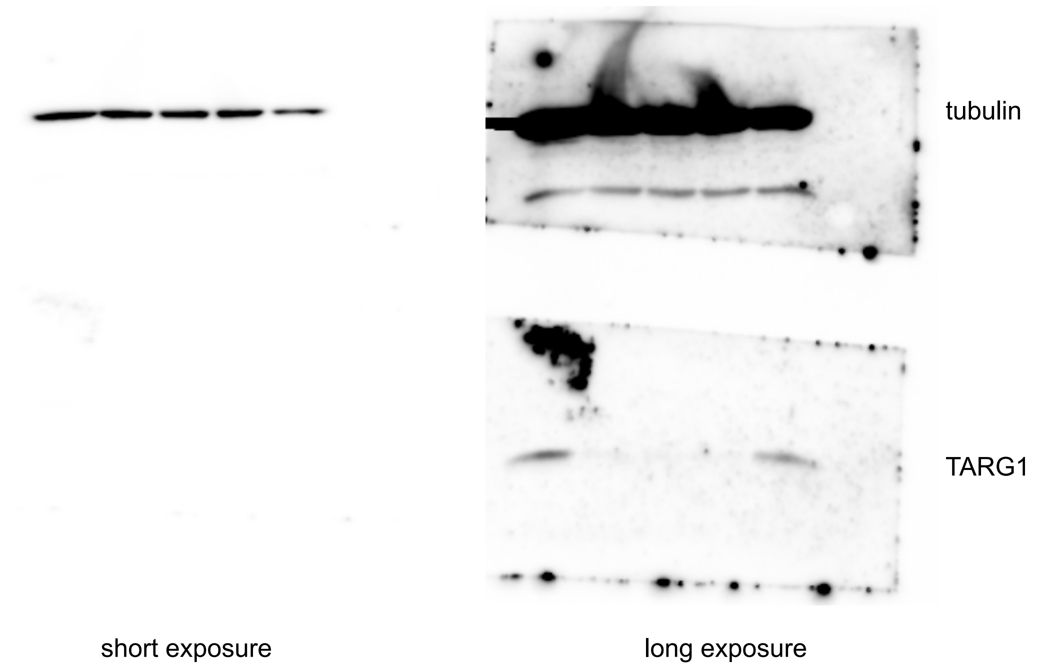

Supplementary Figure 9 Full blots
Belonging to Supplementary Figure S8a and 8b

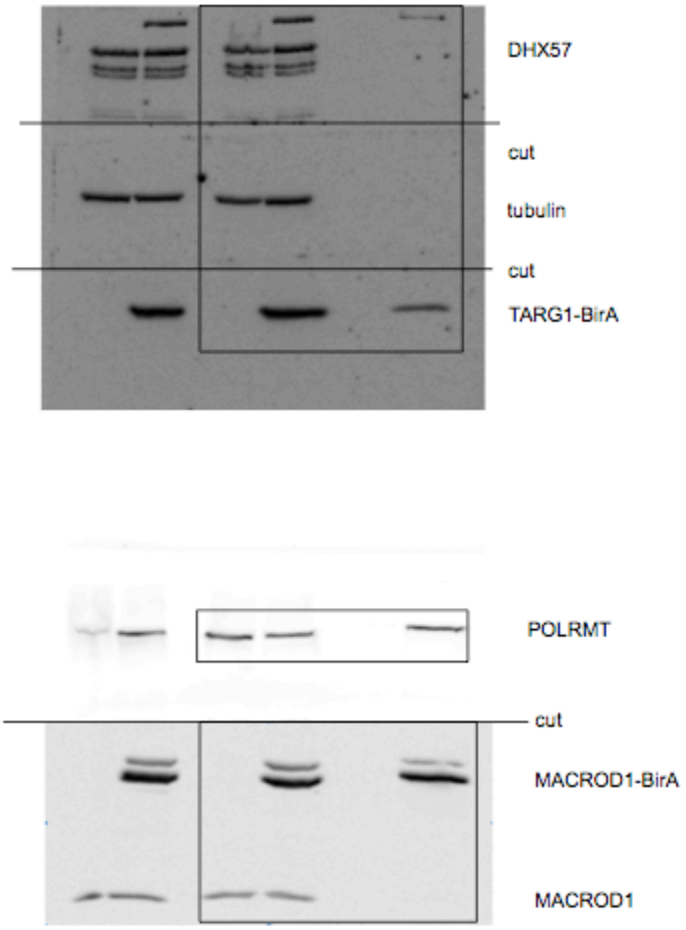

Supplementary table 1: MACROD1, MACROD2 and TARG1 antibody analysis

	Protein	Antibody	Localisation	Size	Validation
**Databases**					
Uniprot	MACROD1	n/a	Nucleus	35.5 kDa	The predicted molecular weight of MACROD1 is higher than the observed size in Western Blot, due to cleavage of the N-terminus
Uniprot	MACROD2	n/a	Nucleus	47 kDa	n/a
Uniprot	TARG1	n/a	Nucleus	17 kDa	n/a
**Published works**					
Agnew, 2017	MACROD1	ab122688	IF: Mitochondria; nucleus	WB: ± 28 kDa; larger bands	Not validated with siRNA on WB; no whole blots shown. Nuclear signal in IF does not disappear with siRNA
Shao, 2018	MACROD1	Santa cruz goat polyclonal	n/a	Whole blots not shown	Not validated; antibody not available anymore
Golia, 2017	MACROD2	Home-made	IF: nucleus; cytoplasm	WB: ± 60 kDa plus larger bands	siRNA; the band at ± 60 kDa disappears after siRNA
Butepage, 2018	TARG1	3A5	IF: unspecific	WB: ± 17	The 17 kDa band disappears after siRNA or knockout
This work	MACROD1	28C11	IF: mitochondria	WB: ± 28 kDa	The 28 kDa band disappears after siRNA treatment
This work	MACROD1	25E9	n/a	WB: ± 28 kDa; ± 39 kDa; ±19 kDa in some lines	The 28 kDa band disappears after siRNA treatment or knockout; the other bands are non-specific
This work	MACROD2	18D12	IF: endogenous not detectable	WB: ± 60 kDa	Not validated with siRNA but confirms previous work by Golia 2017
**Companies**					
HPA www.proteinatlas.org	MACROD1	HPA041031	IF: nucleoplasm	n/a	Not validated
HPA www.proteinatlas.org	MACROD1	HPA071075	n/a	WB: bands at ± 28 kDa and ± 50 kDa depending on cell line	Not validated
HPA www.proteinatlas.org	MACROD2	HPA049076	IF: nucleoli nucleoplasm;	WB: ± 50 kDa	Not validated
HPA www.proteinatlas.org	TARG1	HPA029036	IF: nucleoli; nucleoplasm	WB: ± 56 kDa in some cell lines	Not validated
Abcam	MACROD1	ab157603	n/a	WB: ± 39 kDa and ± 60 kDa	Not validated; data from Abcam website
Abcam	TARG1	ab122032	IF: nucleolus; nucleoplasm	Many large bands according to customer review	Not validated; data from Abcam website
ThermoFisher	MACROD1	PA5-59402	n/a	n/a	Not validated; data from ThermoFisher website
ThermoFisher	MACROD1	PA5-67144	IF: nucleoplasm	n/a	Not validated; data from ThermoFisher website
ThermoFisher	MACROD1	PA5-70915	n/a	n/a	Not validated; data from ThermoFisher website
ThermoFisher	MACROD2	PA5-45950	n/a	± 38 kDa and ± 22 kDa	Not validated; data from ThermoFisher website
ThermoFisher	MACROD2	PA5-106997	n/a	± 60 kDa	Not validated; data from ThermoFisher website
ThermoFisher	TARG1	PA5-56043	IF: nucleus; nucleoli; cytoplasmic dots	n/a	Not validated; data from ThermoFisher website

Supplementary Table S1 MACROD1, MACROD2 and TARG1 antibody analysis
The currently commercially available antibodies, as well as antibodies described in previous publications are summarised: the molecular weight of detected species, localisation using immunofluorescence and validation are listed.

The following tables are available for download as .xlsx files.

Supplementary Table S2 BioID analysis of MACROD1 and TARG1
This table contains a list of the interactors that were identified in the mass spectrometry datasets using BioID for MACROD1 and TARG1. Only those proteins that were enriched at least two-fold over control in two experiments are listed.

Supplementary Table S3 BioID analysis of MACROD1 and TARG1 using more stringent criteria
The interactors identified using BioID for MACROD1 and TARG1 as listed in Supplementary Table S3 were compared. New lists were generated with the interactors which are unique to either dataset by removing all proteins appearing in both.

Supplementary Table S4 List of GO-terms used for the generation of the treemap in Figure 6A
The list of TARG1 interactors as described in Supplementary Table S3 was used for Gene Ontology analysis; the resulting terms and p-values are listed in this table.
